# Effectiveness of stop smoking interventions among adults: protocol for an overview of systematic reviews and an updated systematic review

**DOI:** 10.1186/s13643-018-0928-x

**Published:** 2019-01-19

**Authors:** Mona Hersi, Gregory Traversy, Brett D. Thombs, Andrew Beck, Becky Skidmore, Stéphane Groulx, Eddy Lang, Donna L. Reynolds, Brenda Wilson, Steven L. Bernstein, Peter Selby, Stephanie Johnson-Obaseki, Douglas Manuel, Smita Pakhale, Justin Presseau, Susan Courage, Brian Hutton, Beverley J. Shea, Vivian Welch, Matt Morrow, Julian Little, Adrienne Stevens

**Affiliations:** 1 Knowledge Synthesis Group, Clinical Epidemiology Program, Ottawa Hospital Research Institute, Centre for Practice-Changing Research, 501 Smyth Road, Box 201, Ottawa, Ontario K1H 8L6 Canada; 2 Public Health Agency of Canada, Ottawa, Ontario Canada; 3 Lady Davis Institute of the Jewish General Hospital, Montreal, Quebec Canada; 4 Department of Psychiatry, McGill University, Montreal, Quebec Canada; 5 Department of Community Health Sciences, University of Sherbrooke, Sherbrooke, Quebec Canada; 6 Centre de recherche Charles-Le Moyne – Saguenay–Lac-Saint-Jean sur les innovations en santé (CR-CSIS), Université de Sherbrooke, Quebec, Quebec Canada; 7 University of Calgary Cumming School of Medicine, Calgary, Alberta Canada; 8 Alberta Health Services, Calgary, Alberta Canada; 9 Department of Family and Community Medicine, University of Toronto, Toronto, Ontario Canada; 10 Dalla Lana School of Public Health, University of Toronto, Toronto, Ontario Canada; 11 Division of Community Health and Humanities, Memorial University of Newfoundland, St. John’s, Newfoundland Canada; 12 Department of Emergency Medicine, Yale School of Medicine, New Haven, CT USA; 13 Addictions Division, Centre for Addiction and Mental Health, Toronto, Ontario Canada; 14 Department of Otolaryngology, University of Ottawa, Ottawa, Ontario Canada; 15 The Ottawa Hospital, Ottawa, Ontario Canada; 16 Department of Family Medicine, University of Ottawa, Ottawa, Ontario Canada; 17 Ottawa Hospital Research Institute, Ottawa, Ontario Canada; 18 School of Epidemiology and Public Health, Faculty of Medicine, University of Ottawa, Ottawa, Ontario Canada; 19 Bruyere Research Institute, Ottawa, Ontario Canada; 20 School of Psychology, University of Ottawa, Ottawa, Ontario Canada; 21 Patient representative, Vancouver, British Columbia Canada

**Keywords:** Tobacco, Cessation, Stop smoking, Adults, Systematic review

## Abstract

**Background:**

Tobacco smoking is the leading cause of cancer, preventable death, and disability. Smoking cessation can increase life expectancy by nearly a decade if achieved in the third or fourth decades of life. Various stop smoking interventions are available including pharmacotherapies, electronic cigarettes, behavioural support, and alternative therapies. This protocol outlines an evidence review which will evaluate the benefits and harms of stop smoking interventions in adults.

**Methods:**

The evidence review will consist of two stages. First, an overview of systematic reviews evaluating the benefits and harms of various stop smoking interventions delivered in or referred from the primary care setting will be conducted. The second stage will involve updating a systematic review on electronic cigarettes identified in the overview; randomized controlled trials will be considered for outcomes relating to benefits while randomized controlled trials, non-randomized controlled trials, and comparative observational studies will be considered for evaluating harms. Search strategies will be developed and peer-reviewed by medical information specialists. The search strategy for the updated review on e-cigarettes will be developed using that of the candidate systematic review. The MEDLINE®, PsycINFO, Embase, and the Cochrane Library electronic databases will be searched as of 2008 for the overview of reviews and from the last search date of the selected review for the updated review. Organizational websites and trial registries will be searched for unpublished or ongoing reviews/studies. Two reviewers will independently screen the title and abstracts of citations using the liberal accelerated method. Full-text screening will be performed independently by two reviewers. Extracted data will be verified by a second reviewer. Disagreements regarding full-text screening and data extraction will be resolved by consensus or third-party adjudication. The methodological quality of systematic reviews, risk of bias of randomized and non-randomized trials, and methodological quality of cohort studies will be evaluated using AMSTAR 2, the Cochrane risk of bias tool, and a modified version of the Scottish Intercollegiate Guidelines Network critical appraisal tool, respectively. The GRADE framework will be used to assess the quality of the evidence for outcomes.

**Discussion:**

The evidence review will evaluate the benefits and harms of various stop smoking interventions for adults. Findings will be used to inform a national tobacco cessation guideline by the Canadian Task Force on Preventive Health Care.

**Systematic review registration:**

PROSPERO (CRD42018099691, CRD42018099692)

**Electronic supplementary material:**

The online version of this article (10.1186/s13643-018-0928-x) contains supplementary material, which is available to authorized users.

## Background

### Prevalence and burden of tobacco smoking

In 2012, approximately 45,500 deaths (18% of all deaths in Canada) were attributed to tobacco smoking [1]. Smoking continues to be a leading cause of preventable death and disability [2, 3]. Among smoking-related deaths, most were attributable to cancers, cardiovascular disease, and respiratory diseases [1, 4].

Worldwide, it is estimated that nearly one in seven adults smoke tobacco daily [5]. According to the Canadian Community Health Survey (CCHS), five million (16%) Canadians over the age of 12 years in 2017 smoked tobacco [6]. In Canada, daily or occasional smoking is higher in males (19% versus 13%), particularly among those 20 to 34 years of age (24%) [6]. Among females, smoking is most prevalent in those 50 to 64 years of age (17%) [6]. Higher rates of smoking have been shown in people with lower education (<secondary education: 20%; completion of university: 10%) and lower income (lowest household income: 23%; highest household income: 12%) [7, 8]. The rate of smoking in Indigenous populations is two to three times the national average, ranging from 34 to 53% across First Nations, Métis, and Inuit populations [9]. Studies suggest that smoking rates are also higher in people with substance use disorders and mental health issues [10–12]. Although smoking prevalence has declined overall across Canada, smoking rates vary across the country, with Prince Edward Island reporting the lowest (12%) and Newfoundland and Labrador reporting the highest (20%) rates [13].

Smoking is the leading cause of cancer with evidence linking it to increased risk of several types of cancers including lung, mouth, upper aerodigestive tract, bladder, cervix, colon, and rectum [14]. Smoking also increases the risk of non-malignant respiratory diseases (e.g. chronic obstructive pulmonary disease, tuberculosis), cardiovascular disease (e.g. coronary heart disease, stroke, artherosclerosis, aortic aneurysm, peripheral vascular disease), reproductive issues (e.g. infertility, spontaneous abortion, premature birth, low birth weight), neonatal death, sudden infant death syndrome, early menopause, osteoporosis, and many other chronic health conditions [15–19]. Tobacco smoking using a water pipe or hookah is associated with lung and esophageal cancer as well as infectious diseases due to sharing of the pipe [20–22]. Exposure to second- and third-hand smoke also increases the risk of many diseases including stroke, lung cancer, cervical cancer, respiratory diseases, infections, perinatal and neonatal death, and sudden infant death syndrome [16, 23–26].

Smoking is associated with lower health-related quality of life. Longitudinal data from the Canadian National Population Health Survey found that individuals who smoke tobacco had a lower health-related quality of life compared to those who had never smoked. Smoking cessation was associated with improvement in health-related quality of life. In women, health-related quality of life was similar to those who had never smoked tobacco after 10 years of cessation. In men, it took 20 years of cessation to achieve a health-related quality of life equivalent to those who had never smoked tobacco [27].

In 2012, the total cost of tobacco use in Canada was estimated at $16 billion CDN [1]. This estimate includes both direct (i.e. hospital expenditure, physician care, medications) and indirect (i.e. economic loss associated with premature death and disability) costs which were approximately $6.5 billion and $9.5 billion, respectively [1].

Smoking cessation, defined as quitting or the discontinuation of tobacco smoking, reduces the risk of smoking-related diseases and premature death [3, 28, 29]. Quitting at 30 years of age increases life expectancy by a decade while quitting at 40 and 50 years of age increases expectancy by 9 and 6 years, respectively [30]. For every two individuals who quit smoking tobacco, one will avoid a tobacco-related death [31]. According to the 2017 Canadian Tobacco, Alcohol and Drugs Survey, about 63% of Canadians who reported smoking at some point in their life have successfully quit smoking [13]. Among the 44% of respondents who made an attempt to quit in the past year, 16% made a single attempt while 12% attempted four or more times [13]. In 2017, reducing smoking consumption was the most common cessation method (approximately 63%) among survey respondents, followed by the use of pharmacotherapies (approximately 55%) [13]. Approximately 32% of those who attempted to quit tobacco smoking in 2017 used electronic cigarettes (e-cigarette) as a cessation method [13].

## Stop smoking interventions

### Approved pharmacotherapies

Nicotine replacement therapy (NRT) and cytisine are available over-the-counter while varenicline and bupropion are available by prescription [32]. NRT is the most widely used pharmacotherapy for smoking cessation available over the counter. NRT products administer nicotine thereby reducing withdrawal symptoms and cigarette cravings [33]. It is available in various forms (e.g. patches, chewing gum, lozenges, tablets, buccal spray, and inhalers) and nicotine dosages [34]. Cytisine is a naturally occurring nicotine partial agonist found in the laburnum plant and is pharmacologically similar to varenicline [35]. It is approved as a natural remedy for smoking cessation in Canada [36].

Varenicline and bupropion do not contain nicotine. Varenicline is a nicotine receptor partial agonist that triggers the release of dopamine thereby reducing nicotine withdrawal symptoms and relieving cravings [37]. Varenicline also prevents the stimulating effects of nicotine [38]. Bupropion, the only antidepressant medication approved for smoking cessation [39], is a non-competitive antagonist of nicotinic acetylcholine receptors [40] and also inhibits uptake of dopamine, serotonin, and noradrenaline [41]. Although the mechanism of action is unclear, bupropion may promote cessation by reducing nicotine withdrawal symptoms via inhibition of dopamine and noradrenaline reuptake [42].

### Electronic cigarettes

Electronic cigarettes, also known as e-cigarettes, electronic nicotine (or non-nicotine) delivery systems, or vaporizers, represent another potential intervention strategy by which individuals employ behaviour substitution in their efforts to quit smoking. Most e-cigarettes are battery-operated and are used to inhale a vapour that can contain nicotine and other chemicals such as flavourings, propylene glycol, and/or vegetable glycerin [43, 44]. A heating element within the device releases liquid that is vaporized into a fog or smoke-like cloud [43]. These devices can provide similar behavioural and sensory cues of smoking with no or lower levels of nicotine [44]. There is some evidence to suggest that e-cigarettes significantly reduce exposure to other toxic compounds found in combusted cigarette smoke such as carbon monoxide, acrolein, acetaldehyde, and formaldehyde [45, 46]. However, other studies have found that some e-cigarette brands contain high levels of toxic metals including nickel, cadmium, chromium, lead, and manganese [47]. The recently passed Canadian Tobacco and Vaping Products Act (Bill S-5) now allows adults to legally purchase e-cigarettes containing nicotine in Canada. However, it bans the sale of e-cigarettes to individuals under 18 years of age, specific flavours that are appealing to youth (e.g. confectionary, soft drink), ingredients that suggest health benefits (e.g. vitamin, caffeine), and certain types of advertising and promotion (e.g. health benefits, products using tobacco brands) [48].

### Behavioural therapies

There are various behavioural interventions used for tobacco cessation. Broadly, behavioural interventions may promote smoking cessation directly, be directed to improve adherence to smoking cessation pharmacotherapies, or promote other health behaviour change along with the stopping smoking behaviour (e.g. healthy eating, alcohol reduction).

Behavioural interventions can be classified by intensity (very brief, brief, intensive), frequency of contact, modality of contact, type of provider, and content. These factors can influence the effectiveness of the intervention. Details on the specific behavioural change technique(s) (i.e. the content or “the smallest active ingredients of interventions capable of inducing change in behaviour” [49]) that are being targeted are essential in determining not only what components of behaviour support systems are effective, but how they can be replicated in practice [49]. A taxonomy of behavioural change techniques used in individual behavioural support for smoking cessation has been developed to support such evaluations [50]. Examples of behavioural change techniques include goal setting (e.g. setting a quit date), advice on altering routines to avoid exposure to smoking cues, and providing information regarding withdrawal symptoms [50].

Another aspect of behavioural change interventions is understanding the psychological theory underpinning the design of the intervention. For example, the Transtheoretical Model of Change, also known as the ‘Stages of Change’ model, is highly used in the smoking cessation literature, but not supported empirically in systematic review evaluations [51, 52]. Although these theories may have face validity, evaluating them is important not only to understand effectiveness but also to avoid harms. Evidence suggests that stage-based approaches for smoking cessation are not more effective than non-stage interventions indicating that readiness or motivation to stop smoking may not be integral for quitting [51, 52]. Further, stage-based interventions might prevent providers from offering effective treatment to those deemed unmotivated to stop smoking thereby prolonging their exposure to the toxic constituents of smoke.

Brief advice interventions consist of healthcare professionals providing verbal instructions with a “stop smoking message” [53]. These interventions may vary in intensity, frequency, and duration but generally only last a few minutes. Individual or group therapies are led by counsellors such as physicians, nurses, clinical psychologists, and counsellors. The objective of such interventions is to provide an opportunity for people who smoke to share cessation experiences; derive support; learn coping skills to manage cravings, lapses, and relapses; and promote self-control [54]. More intensive face-to-face interventions require greater effort and resources and may only reach a small segment of the smoking population [55]. Telephone counselling can supplement or replace these therapies as a way of providing services to a larger number of people [56]. These can take the form of proactive (i.e. counsellor-initiated) or reactive counselling (i.e. tobacco smoker-initiated) [57].

Self-help interventions are information aids, such as manuals or programmes, used by individuals without the direct support of healthcare professionals [55]. The goal is to provide some of the benefits of brief advice and counselling but without the necessary attendance. Traditional self-help materials, such as print, audio, and video recordings, can be more widely accessible and are increasing their reach via newer technology (e.g. web-based, mobile applications and games, streaming content) [58]. However, increased reach may not necessarily be more effective if the content of the instruction is not effective.

### Exercise

Some therapies, such as exercise-based interventions, have been used alone or as adjuncts to other interventions. Exercise alleviates withdrawal symptoms and relieves cravings [59]. Although the mechanism of action is unclear, several hypotheses have been proposed [59, 60]. The biological hypothesis suggests that exercise and nicotine have similar impacts on beta-endorphins, cortisol, noradrenaline, and adrenaline [59, 60]. For example, like nicotine, exercise stimulates the release of adrenaline and noradrenaline thereby relieving cravings [59]. Although the evidence is inconsistent, the beneficial effect of exercise on cessation may also be attributed to increases in positive affect or distraction from withdrawal symptoms and cravings [59, 60].

### Alternative therapies

Alternative therapies for smoking cessation include hypnosis, acupuncture (including acupressure and electrostimulation), and laser therapy [59, 61]. It is hypothesized that acupuncture, acupressure, and laser therapy alleviate withdrawal symptoms by stimulating peripheral nerves which triggers release of opioid peptides, dopamine, enkephalin, and serotonin [62]. The mechanism of action underpinning the effect of hypnotherapy on smoking cessation is related to strengthening impulse control [63]. St. John’s Wort is a herbal product commonly used by patients as an alternative to standard antidepressant medications [64]. St. John’s Wort may promote smoking cessation by alleviating tobacco withdrawal symptoms and decreasing negative affect through various mechanisms including inhibition of monoamine oxidase A and B and dopamine and noradrenaline reuptake [39, 65]. S-Adenosylmethionine (SAMe), a natural health product, promotes the production of dopamine and norepinephrine and may therefore alleviate tobacco withdrawal symptoms [66].

## Current clinical practice and recommendations

### Canadian guidelines

In 2011, the Canadian Action Network for the Advancement, Dissemination and Adoption of Practice-informed Tobacco Treatment (CAN-ADAPTT) published recommendations for adults and specific populations (e.g. Indigenous, hospital-based, mental health, substance use disorders, pregnant and breastfeeding women, and youth) that were informed by six guidelines [67]. CAN-ADAPTT recommends that healthcare providers routinely ask patients about their tobacco use and advise those who smoke tobacco to quit. Those willing to begin treatment should be offered assistance such as brief advice, individual and group counselling (focused on problem-solving skills or skills training and providing support), self-help materials, motivational interviewing, or pharmacotherapies. Where possible, CAN-ADAPTT recommends combining counselling and pharmacotherapies as the preferred approach. Providers are encouraged to follow-up regularly and modify treatment as needed.

The Registered Nurses’ Association of Ontario (2017) released recommendations based on previous guidelines and a systematic review [68]. They recommend using brief interventions to screen individuals for tobacco use, developing person-centered tobacco intervention plans, referring tobacco users to intensive interventions and counselling on the use of pharmacotherapies (i.e. NRT, varenicline, bupropion), and evaluating the effectiveness of these interventions and adjusting as needed. They conclude that there is insufficient evidence regarding e-cigarettes, hypnotherapy, laser therapy, electrostimulation, acupressure, and acupuncture as cessation tools. For pregnant or postpartum women, they recommended intensive behavioural counselling, in conjunction with NRT.

### Guidelines from international organizations

Guidelines from international organizations are consistent in recommending behavioural interventions and/or pharmacotherapies (i.e. NRT, bupropion, and varenicline) for smoking cessation. The UK National Institute for Health and Care Excellence (NICE, 2018) recommends individual or group behavioural support, very brief advice, bupropion, combination of short- and long-acting NRT, or varenicline in conjunction with behavioural support [69]. New Zealand’s Ministry of Health (2014) recommends brief advice (approximately 30 s), behavioural support, NRT, buproprion, varenicline, and nortriptyline. They consider a combination of behavioural and pharmacotherapy to be the most effective [70]. As part of their “Risk estimation and the prevention of cardiovascular disease” guideline, the Scottish Intercollegiate Guidelines Network (2017) recommends (1) varenicline or combination NRT (i.e. “interventions involving more than one type of nicotine replacement delivery”) alone or as part of a smoking cessation programme, and (2) bupropion and single NRT [71]. The US Preventive Services Task Force is currently updating their 2015 guideline [17]. The 2015 guideline, based on an overview of reviews [72], recommends behavioural interventions and approved pharmacotherapies (i.e. bupropion, varenicline, NRT). Only behavioural interventions are recommended for pregnant women as the evidence regarding pharmacotherapies was insufficient for this subgroup.

We did not identify any guideline that recommends the use of e-cigarettes for smoking cessation. However, NICE recommends that, when advising those interested in using e-cigarettes containing nicotine, primary health care providers should communicate that “many people have found them helpful to quit smoking cigarettes” and that e-cigarettes, while not without risk, are less harmful than tobacco smoking [69]. Similarly, Public Health England’s recently developed guidance for clinicians includes e-cigarettes as a smoking cessation option to discuss with patients. The guidance indicates that e-cigarettes present less risk than smoking and that they may be as or more effective than nicotine replacement therapy [73]. Other organizations state that there is currently insufficient evidence regarding the beneficial effects of e-cigarettes to make recommendations [17, 71].

A majority of the available guidelines are out of date (i.e. last database search range: 2008 to 2015). Although recent, the NICE guideline excludes several smoking cessation interventions including varenicline, exercise, and alternative therapies (e.g. acupuncture, hypnotherapy) [69]. Limitations in existing clinical practice guidelines necessitate the development of a Canadian guideline on tobacco cessation strategies for adults.

## Objective and key questions

The goal of this evidence review is to determine the effectiveness of stop smoking strategies for adults. Pharmacotherapy, behaviour change interventions, electronic cigarettes, exercise interventions, and complementary and alternative medicine interventions will be considered. Adult populations will include subgroups of interest such as those with co-morbid conditions, pregnant women, various demographic factors, and the distinction of opportunistic and treatment-seeking individuals. This synthesis will be used by the Canadian Task Force on Preventive Health Care (Task Force) to inform their development of a clinical practice guideline on stop smoking interventions.

The evidence review will consist of two stages. First, the overview of stop smoking interventions will be conducted. An overview of systematic reviews approach was selected to compile the evidence base in light of the large volume of primary and synthesized evidence that exists. The second stage will involve updating the most recent, comprehensive, and high-quality systematic review on e-cigarettes identified in the overview of reviews. Only the e-cigarettes strategy will be updated because of the increasing use of this strategy and its quickly evolving evidence base. This protocol document serves to outline the methodology for both review types.

For the purpose of the evidence review, tobacco smoking will refer to any form of smoked tobacco (e.g. cigarettes, pipes, cigars, cigarillos, via water pipe or hookah). This will not include tobacco use for traditional or ceremonial purposes such as that used by Indigenous people in sacred rituals and prayers for healing and purification [74, 75].

### Stage 1: Overview of systematic reviews of stop smoking interventions

**Fig. 1:**
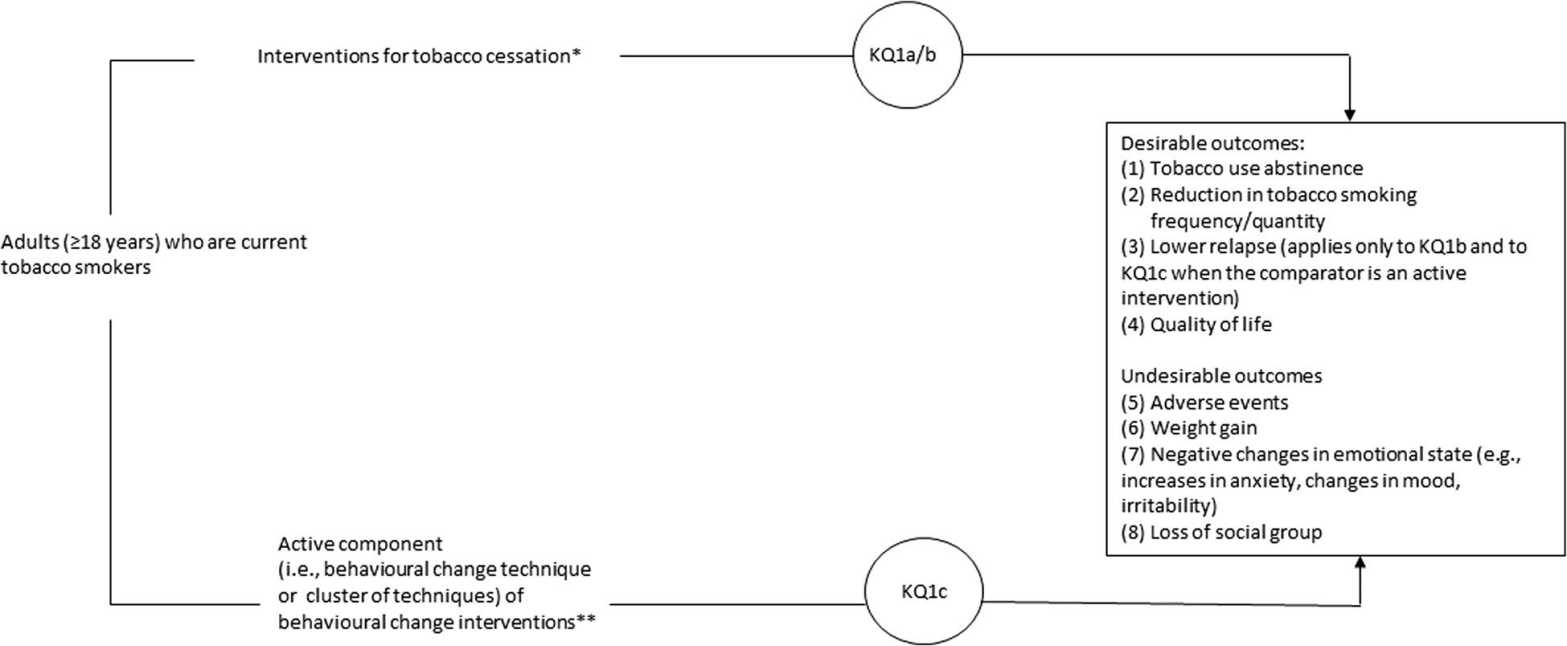
Analytic framework for the overview of reviews. *Practitioner advice (of varying length/intensity, and by various provider types); Intensive individual counselling (of varying length, of varying number of sessions, and by various provider types); Intensive group counselling (of varying length, of varying number of sessions, and by various provider types); Self-help interventions (print-based or web-/computer-based); Internet or computer-based interventions with counselling/support; Telephone-based interventions (e.g., mobile phone-based, quit lines/help lines) with counselling/support; Nicotine receptor partial agonists (varenicline and cytisine); Bupropion; Nicotine replacement therapy (e.g., patch, gum, lozenge, mist, inhaler); Ecigarettes; Exercise interventions; ‘Alternative’ therapies (e.g., acupuncture, acupressure, electrostimulation, hypnosis, St. John’s Wort, S-adenosylmethionine); Combinations of interventions. **Practitioner advice (of varying length/intensity, and by various provider types); Intensive individual counselling (of varying length, of varying number of sessions, and by various provider types); Intensive group counselling (of varying length, of varying number of sessions, and by various provider types); Self-help interventions (print-based or web-/computer-based); Internet or computer-based interventions with counselling/support; Telephone-based interventions (e.g., mobile phone-based, quit lines/help lines) with counselling/support; Other behaviour change interventions evaluated on a case-by-case basis with the Working Group

The overview will evaluate the benefits and harms of stop smoking interventions among adults. If feasible, the overview will also evaluate the benefits and harms of behavioural change techniques (i.e. “the smallest active ingredients of interventions capable of inducing change in behaviour” [49]). Figure 1 illustrates the framework of the overview of systematic reviews. The overview will address the following key questions:

*Key question 1a* (*KQ1a*). What are the benefits and harms of interventions to promote cessation of tobacco smoking among adults?

*Key question 1b* (*KQ1b*). What is the comparative effectiveness (benefits and harms) of interventions to promote cessation of tobacco smoking among adults?

*Key question 1c* (*KQ1c*). What are the benefits and harms of behavioural change techniques or clusters of techniques to promote cessation of tobacco smoking among adults?

### Stage 2: Updated systematic review on e-cigarette use for smoking cessation

This update will evaluate the benefit and harms of e-cigarettes to promote cessation of tobacco smoking among adults. This protocol outlines key questions and eligibility criteria for the updated review. However, should the candidate review from which to update have slightly different parameters, we will transparently declare any necessary changes from the protocol in the final report.

*Key question 2a* (*KQ2a*). What are the benefits and harms of electronic cigarettes for tobacco smoking cessation in adults?

*Key question 2b* (*KQ2b*). What is the comparative effectiveness (benefits and harms) of electronic cigarettes for tobacco smoking cessation in adults?

## Methods

The evidence review will be completed by the Evidence Review and Synthesis Centre (ERSC) at the Ottawa Hospital Research Institute. A working group (WG) of Task Force members and external content experts was formed for development of the topic, refinement of the key questions and scope, and rating of outcomes. Outcomes were rated on a scale of 1 to 9 according to the Grading of Recommendations Assessment, Development and Evaluation (GRADE) methodology; those rated as critical (mean score 7 to 9) and important (mean score 4 to 6) for decision-making were selected. Patients identified through patient engagement activities conducted by the St. Michael’s Hospital Knowledge Translation Program have also rated the outcomes. The process of incorporating patient priorities is described in the CTFPHC’s Patient Engagement Protocol (https://canadiantaskforce.ca/methods/patient-preferences-protocol/).

Reporting of this protocol was guided by the PRISMA Statement for Protocols (PRISMA-P) to the extent possible and where appropriate [76] (Additional file 1). The protocol is registered in PROSPERO (https://www.crd.york.ac.uk/PROSPERO/) (CRD42018099691, CRD42018099692). The final overview will be reported using the Preferred Reporting Items for Overviews of systematic reviews including harms pilot checklist (PRIO-harms) [77], and the updated systematic review will be reported using PRISMA [78].

A team of clinical and content experts will be consulted at key points during the conduct of the evidence review. Amendments to this protocol will be noted in the final report.

### Stage 1: Overview of systematic reviews of stop smoking interventions

Guidelines for the conduct of overviews of reviews are currently lacking [79]. Given this current gap, the methodology for this overview will be guided by the *Cochrane Handbook of Systematic Reviews of Interventions* (*Chapter 22*) [80] as well as other available reports on overview methodology [79, 81–85].

#### Literature search

The search strategy will be developed and tested through an iterative process by an experienced medical information specialist in consultation with the review team. We will search Ovid MEDLINE®, Ovid MEDLINE® Epub Ahead of Print, In-Process & Other Non-Indexed Citations, PsycINFO, Embase Classic + Embase, and the Cochrane Library on Wiley. Databases will be searched from 2008 to the current date. The draft search strategy can be found in Additional file 2. The search strategy will be peer-reviewed using the PRESS 2015 guideline [86]. Results of the PRESS reviews will be provided in an appendix in the final report.

We will search for unpublished literature and reports of ongoing and completed reports using the Canadian Agency for Drugs and Technologies in Health (CADTH) Grey Matters checklist [87] and through searches of the following websites: CADTH, Ontario Tobacco Research Unit, The Canadian Partnership Against Cancer (cancerview.ca), SurgeonGeneral.gov, Philip Morris, Foundation for a Smoke-free World, Public Health England, Tobacco.org, Truth Initiative, Physicians for a Smoke-Free Canada, Centers for Disease Control and Prevention Smoking and Health Resource Library, Canadian Cancer Society, American Cancer Society, American Thoracic Society, US National Cancer Institute, US National Comprehensive Cancer Network, National Institute for Health and Care Excellence, World Health Organization Framework Convention on Tobacco Control, World Health Organization’s International Clinical Trials Registry Platform, OpenTrials.net, International Prevention Research Institute, North American Quitline Consortium website, and the Ottawa Heart Institute’s Ottawa Model for Smoking Cessation. We will also scan the bibliographies of relevant reviews and other identified overviews for grey literature and references not identified in our database search. Grey literature searching will be restricted to English and French language documents and will be limited to what can be completed within 1 week by one reviewer.

#### Eligibility criteria

KQ1a and KQ1b will examine interventions that can be delivered or referred to in the primary care setting. This includes certain behavioural change interventions, pharmacotherapies, e-cigarettes, exercise interventions, and alternative therapies (Table 1). Interventions that cannot be delivered or referred to by a wide variety of primary care practitioners (e.g. quit-to-win contests, biomedical risk assessment, aversive smoking, incentivized cessation) as well as specific behavioural counselling techniques (e.g. motivational interviewing, stage of change-based counselling) which require specialized training that has been shown to vary [88] and may not be readily available to all primary care practitioners will be excluded. We will also exclude reviews on broader public health interventions (e.g. mass media, taxation, packaging restrictions) as well as those on broad lifestyle interventions not specific to tobacco smoking behaviour and that do not attempt to isolate for the effect of our included interventions (i.e. when delivered as part of a multifaceted lifestyle intervention). Generally, pharmacotherapies that are not approved by Health Canada as smoking cessation aids (e.g. clonidine, lobeline, anxiolytics, nortriptyline, opioid antagonists, silver acetate, rimonabant) or not available in Canada (e.g. Nicobrevin, Nicobloc, nicotine vaccines, mecamylamine) will be excluded. However, due to their ease of access, an exception will be made for St. John’s Wort (sold in various forms in pharmacies and health stores across Canada), cytisine, and S-adenosylmethionine (SAMe) (licensed natural health products).

**Table 1:** Inclusion and exclusion criteria for key question 1a and 1b

“PICO” structured question element	Inclusion	Exclusion
Population	KQ1a/b: adults (≥ 18 years) who are current tobacco smokers (as defined by a given study/review) The overview of reviews will seek information on various population groups: • Fewer versus more quit attempts • Opportunistic versus individuals seeking treatment • Baseline level of nicotine dependence (e.g. using a validated scale or cigarettes per day as a proxy) • By demographic factors (age, SES, sex, ethnicity, LGBTQ+) • By comorbid conditions (e.g. mental illness, HIV infection, cardiovascular disease, COPD, obesity, substance use disorder) • By pregnancy status	▪ Reviews exclusively in children/adolescents (i.e. under 18 years old) ▪ Studies that involve interventions targeted to adults other than the tobacco smoker (e.g. partners, healthcare providers)
Intervention	KQ1a/b: interventions to promote abrupt (i.e. “all at once”) or gradual (reducing smoking to quit) tobacco smoking cessation that can be directly delivered or referred to by primary care practitioners and are available in Canada^a^ • Practitioner advice (of varying length/intensity, and by various provider types) o Very brief/minimal advice (as defined by a given review) o Brief advice (as defined by a given review) • Intensive individual counselling (of varying length, of varying number of sessions, and by various provider types) • Intensive group counselling (of varying length, of varying number of sessions, and by various provider types) • Self-help interventions^c^(print-based or web/computer-based) • Internet or computer-based interventions with counselling/support^c^ • Telephone-based interventions (e.g. mobile phone-based, quit lines/help lines) with counselling/support ^c^ • Nicotine receptor partial agonists (varenicline and cytisine^d^) • Bupropion • Nicotine replacement therapy^e^(e.g. patch, gum, lozenge, mist, inhaler) • E-cigarettes^f^ • Exercise interventions • “Alternative” therapies (e.g. acupuncture, acupressure, electrostimulation, hypnosis, St. John’s Wort^d^, S-adenosylmethionine^d^) • Combinations of interventions Other interventions encountered in the literature will be assessed on a case-by-case basis in consultation with the WG. The overview of reviews will seek information on the effects of variations in the delivery of stop smoking interventions (e.g. dose, duration of intervention, number of session)	Interventions that cannot feasibly or readily be delivered or referred to by a wide variety of primary care practitioners: • Quit-to-win contests • Biomedical risk assessment • Aversive smoking (e.g. rapid smoking) • Incentivized cessation Reviews that focus solely on specialized behavioural counselling interventions (e.g. motivational interviewing, stage of change-based interventions).^b^ Pharmacotherapies that are not approved by Health Canada as smoking cessation aids (e.g. clonidine, lobeline, anxiolytics, nortriptyline, opioid antagonists, silver acetate, rimonabant) or not available in Canada (e.g. Nicobrevin, Nicobloc, nicotine vaccines, mecamylamine) Broader public health interventions (e.g. mass media, taxation, packaging restrictions) Reviews on broad lifestyle interventions not specific to tobacco smoking behaviour and that do not attempt to isolate for the effect of our included interventions (when delivered as part of a multifaceted lifestyle intervention, for example)
Comparator	KQ1a: ▪ Placebo ▪ No intervention ▪ Usual care ▪ Waitlist ▪ Minimal intervention KQ1b: ▪ Other intervention (e.g. head-to-head comparisons, comparisons of types or intensities of advice/counselling) ▪ Other combination of interventions ▪ The same intervention, but used to promote cessation by reducing smoking to quit as opposed to quitting abruptly or vice versa	
Outcomes	Critical • Tobacco use abstinence (as defined in a given review) Important • Reduction in tobacco smoking frequency/quantity • Relapse (*KQ1b only*)^g^ • Quality of life (using validated scales) • Adverse events (as defined in a given review) • Possible adverse outcomes: o Weight gain o Changes in emotional state (e.g. increases in anxiety, changes in mood, irritability) o Loss of social group^h^	
Timing of outcome assessment	For abstinence/relapse, and quality of life outcomes: ▪ Minimum 6 months from quit date (if reported) or from initiation of intervention (if quit date not specified) All other outcomes: Any point after initiation of intervention	
Setting	▪ Reviews in which some or all of the included studies are in settings that could serve as the primary point of contact for individuals to receive smoking cessation advice, including: • Family medicine clinics • Walk-in clinics • Smoking cessation clinics • Urgent care facilities • Emergency departments • Public health units • Pharmacies • Dental offices • Behavioural health/substance use treatment facilities (ambulatory or outpatient) • Telehealth • Academic research settings The effect of various settings may be examined	▪ Reviews exclusively in settings not relevant to primary care including workplaces, schools, inpatient settings, and medical specialist settings ▪ Reviews in which > 50% of included studies took place in countries “high”, “medium”, or “low” on the Human Development Index http://hdr.undp.org/en/composite/HDI
Study design	Systematic^i^ reviews Overviews^j^ of systematic^i^ reviews that include a network meta-analysis	• Primary studies • Editorials • Commentaries
Language	▪ English ▪ French	
Dates of publications	2008 to present	

^a^In this context, primary care practitioners refer to the provider of first contact for the delivery or referral to stop smoking interventions. This could include physicians, nurses, pharmacists, oral health professionals, counsellors, etc.

^b^Reviews examining specialized behavioural counselling interventions will be excluded, as the target audience for this guideline is primary care. These interventions require specialized training, the amount of which has been shown to vary but can be substantial [88] and may not be readily available for many primary care practitioners

^c^We define “self-help interventions” to include “any manual or programme to be used by individuals to assist a quit attempt not aided by health professionals, counsellors or group support” as per the definition in Hartmann-Boyce et al. [55]. This differs from interventions that utilize computers, the web, or mobile phones to deliver interventions that involve counselling/support, although the platform of delivery may be the same

^d^Certain products are relevant for inclusion despite not being approved for use as smoking cessation aids by Health Canada, due to their ease of access. These include St. John’s wort (sold in various forms in pharmacies and health stores across Canada), cytisine, and S-adenosylmethionine (licensed natural health products)

^e^Patches, gums, mists/sprays, and inhalers are the available forms of NRT in Canada

^f^The practice of using e-cigarettes (“vaping”; including e-cigarettes with nicotine) is increasingly popular, with use being higher among tobacco smokers [89]. Data from the CDC suggest that it was the most commonly used method to quit smoking in 2014–2016 after simply giving up cigarettes all at once or gradually cutting back [90]. The massive interest in these products from the public and tobacco smokers, as well as the evolving evidence base surrounding them, makes them essential to include

^g^The outcome “relapse” was initially considered critical based on WG rating. However, based on discussion with WG members it was decided that this outcome should be considered important. It was also decided that this outcome is most important for KQ1b

^h^Although initially rated as being of limited importance by the WG, based on discussions with WG members, it was decided that this outcome should be considered as important. Clinical experts and patients rated this outcome as important

^i^Reviews will be considered systematic if they meet the four following criteria: (1) searches at least one database, (2) reports their selection criteria, (3) conducts quality or risk of bias assessment on included studies, and (4) provides a list and synthesis of included studies

^j^Overviews will included if they meet the following criteria: (1) search at least one database, (2) report their selection criteria and how they will handle the inclusion of overlapping reviews, (3) provide information on the quality or risk of bias assessment of studies included in reviews, (4) provide a list of relevant reviews, (5) report the synthesized evidence from the included reviews, and (6) explicit declaration that the decision to undertake the network meta-analysis was made with firsthand knowledge of the primary studies, to ensure appropriateness of the analysis

Systematic reviews for KQ1a and KQ1b will be selected for inclusion according to the eligibility criteria outlined in Table 1 [89, 90].

In addition to the other interventions listed in Table 1, the intent of KQ1a/b is to capture reviews which examine behavioural change *interventions* (e.g. practitioner advice, counselling, self-help interventions). These reviews may provide information on the active components of these interventions, referred to as behavioural change *techniques*. Examples of such techniques include providing information on consequences of smoking, explaining the importance of abrupt cessation, strengthening ex-smoker identity, and receiving prompt commitment from the patient [50]. If there is sufficient data, subgroup analysis by behavioural change technique or clusters of techniques will be performed for KQ1a/b (see the “Subgroup analysis” section).

While the intent of KQ1a/b is to synthesize reviews of behavioural change intervention*s* (these reviews may or may not report the behavioural change techniques used as part of these interventions), the intent of KQ1c is to capture reviews which specifically examine the effectiveness of behavioural change techniques or cluster of techniques. A taxonomy of behavioural change techniques used in smoking cessation interventions will guide the coding of techniques encountered in the literature [50].

Eligibility of reviews for KQ1c will be evaluated in consultation with the WG on a case-by-case basis with selection for inclusion dependent on applicability to the primary care setting. For example, the WG may decide to include behavioural change interventions outside of those listed in Table 2 or may decide to include reviews in specialty settings if the review examines behavioural change techniques that can reasonably be applied in primary care. Selection of reviews for KQ1c will be guided by the eligibility criteria outlined in Table 2. All decisions regarding the selection of reviews will be reported in the completed review.

**Table 2:** Inclusion and exclusion criteria for Key Question 1c

“PICO” structured question element	Inclusion	Exclusion
Population	Adults (≥ 18 years) who are current tobacco smokers (as defined by a given study/review) The overview of reviews will seek information on various population groups: • Fewer versus more quit attempts • Opportunistic versus individuals seeking treatment • Baseline level of nicotine dependence (e.g. using a validated scale or cigarettes per day as a proxy) • By demographic factors (age, SES, sex, ethnicity, LGBTQ+) • By comorbid conditions (e.g. mental illness, HIV infection, cardiovascular disease, COPD, obesity, substance use disorder) • By pregnancy status	▪ Reviews exclusively in children/adolescents (i.e. under 18 years old) ▪ Studies that involve interventions targeted to adults other than the tobacco smoker (e.g. partners, healthcare providers)
Intervention	Interventions to promote abrupt (i.e. “all at once”) or gradual (reducing smoking to quit) tobacco smoking cessation that can be directly delivered or referred to by primary care practitioners and are available in Canada^a^ We will seek reviews which specifically examine the effectiveness of behavioural change techniques or cluster of techniques (e.g. explaining the consequences of smoking, strengthening ex-tobacco smoker identity, explaining the importance of abrupt cessation) which may be used as a component of the following behavioural change interventions: • Practitioner advice (of varying length/intensity, and by various provider types) o Very brief/minimal advice (as defined by a given review) o Brief advice (as defined by a given review) • Intensive individual counselling (of varying length, of varying number of sessions, and by various provider types) • Intensive group counselling (of varying length, of varying number of sessions, and by various provider types) • Self-help interventions^b^ (print-based or web/computer-based) • Internet or computer-based interventions with counselling/support^b^ • Telephone-based interventions (e.g. mobile phone-based, quit lines/help lines) with counselling/support^b^ • Combinations of interventions Behavioural change techniques delivered as part of other behavioural change interventions (i.e. other than those listed above) will be assessed on a case-by-case basis in consultation with the working group. We will seek information on intervention characteristics which may moderate the effectiveness of behavioural change techniques (e.g. duration of intervention, number of sessions)	Reviews which intend to examine behavioural change interventions rather than behavioural change techniques.
Comparator	▪ No intervention ▪ Usual care ▪ Waitlist ▪ Minimal intervention Behavioural change techniques or cluster of techniques delivered as part of: ▪ Other behavioural change intervention (e.g. head-to-head comparisons, comparisons of types or intensities of advice/counselling) ▪ Other combination of behavioural change interventions ▪ The same behavioural change intervention, but used to promote cessation by reducing smoking to quit as opposed to quitting abruptly or vice versa	
Outcomes	Critical • Tobacco use abstinence (as defined in a given review) Important • Reduction in tobacco smoking frequency/quantity • Relapse (only when the comparator is an active intervention)^c^ • Quality of life (using validated scales) • Adverse events (as defined in a given review) • Possible adverse outcomes: o Weight gain o Changes in emotional state (e.g. increases in anxiety, changes in mood, irritability) o Loss of social group^d^	
Timing of outcome assessment	For abstinence/relapse, and quality of life outcomes: ▪ Minimum 6 months from quit date (if reported) or from initiation of intervention (if quit date not specified) All other outcomes: Any point after initiation of intervention	
Setting	Settings that could serve as the primary point of contact for individuals to receive smoking cessation advice, including: • Family medicine clinics • Walk-in clinics • Smoking cessation clinics • Urgent care facilities • Emergency departments • Public health units • Pharmacies • Dental offices • Behavioural health/substance use treatment facilities (ambulatory or outpatient) • Telehealth • Academic research settings Reviews in other settings (e.g. inpatient or specialist medical settings) will be assessed on a case-by-case basis in consultation with the working group The effect of various settings may be examined	▪ Reviews in which > 50% of included studies took place in countries “high”, “medium”, or “low” on the Human Development Index http://hdr.undp.org/en/composite/HDI
Study design	Systematic^e^ reviews Overviews^6^ of systematic^e^ reviews that include a network meta-analysis	• Primary studies • Editorials • Commentaries
Language	▪ English ▪ French	
Dates of publications	2008 to present	

^a^In this context, primary care practitioners refer to the provider of first contact for the delivery or referral to stop smoking interventions. This could include physicians, nurses, pharmacists, oral health professionals, counsellors, etc.

^b^We define “self-help interventions” to include “any manual or programme to be used by individuals to assist a quit attempt not aided by health professionals, counsellors or group support” as per the definition in Hartmann-Boyce et al. [55]. This differs from interventions that utilize computers, the web, or mobile phones to deliver interventions that involve counselling/support, although the platform of delivery may be the same

^c^The outcome “relapse” was initially considered critical based on WG rating. However, based on discussion with WG members, it was decided that this outcome should be considered important. It was also decided that this outcome is most important for head-to-head comparisons. We will only collect data for this outcome when the comparator is an active intervention such as behavioural change techniques or cluster of techniques delivered as part of a behavioural change intervention different from that offered to the intervention group (e.g. behavioural change technique or cluster of techniques delivered as part of practitioner advice versus intensive individual counselling)

^d^Although initially rated as being of limited importance by the WG, based on discussions with WG members, it was decided that this outcome should be considered as important. Clinical experts and patients rated this outcome as important

^e^Reviews will be considered systematic if they meet the four following criteria: (1) searches at least one database, (2) reports their selection criteria, (3) conducts quality or risk of bias assessment on included studies, and (4) provides a list and synthesis of included studies

^6^Overviews will included if they meet the following criteria: (1) search at least one database, (2) report their selection criteria and how they will handle the inclusion of overlapping reviews, (3) provide information on the quality or risk of bias assessment of studies included in reviews, (4) provide a list of relevant reviews, (5) report the synthesized evidence from the included reviews, and (6) explicit declaration that the decision to undertake the network meta-analysis was made with firsthand knowledge of the primary studies, to ensure appropriateness of the analysis

#### Study selection

Duplicates will be identified and removed using Reference Manager [91]. Title and abstract and full-text screening will be conducted using an online systematic review managing software, Distiller Systematic Review (DistillerSR) Software© [92]. Two reviewers will independently screen the title and abstracts of citations using the liberal accelerated method (i.e. a second reviewer verifies records excluded by a first reviewer). References will be randomized, and screening will be done concurrently to ensure that each reviewer cannot determine whether a given reference was excluded by another reviewer. The full text of potentially relevant citations will be retrieved, and two reviewers will independently assess the article for relevancy. If unclear whether a review is eligible after duplicate review, a third person will be consulted before excluding the review. Conflicts will be resolved by consensus or by consulting with a third team member. The reasons for exclusion at full-text screening will be documented.

Both screening forms will be piloted by reviewers prior to commencement of screening, with adjustments made, as needed, to maximize efficiency. If necessary, articles will be ordered via interlibrary loan. Only those received within 30 days will be included. Exclusions due to unavailability of articles will be noted.

A list of potentially relevant reviews available only in abstract form will be made available, but these studies will not be included in the overview.

#### Data mapping and overlap detection

Given the proliferation of systematic reviews [81], we anticipate that we will encounter multiple systematic reviews covering the same research question (i.e. population, intervention, comparison, outcomes, time points, and settings). Such reviews are expected to rely on the same evidence base (i.e. same studies and data); therefore, inclusion of these overlapping systematic reviews may potentially bias the overview findings as the same primary studies are counted more than once [93].

While there is currently no optimal approach for addressing the issue of overlapping reviews [79], existing options include the following: (1) limiting inclusion to a single systematic review using a priori established criteria or (2) including all available reviews and computing the degree of overlap [79, 81, 93]. Limiting inclusion to a single systematic review for a given research question may result in missing data, and while inclusion of all available reviews may improve comprehensiveness, it also increases workload and complexity [81].

To detect and address overlapping systematic reviews, we will first map the research questions (i.e. population, intervention, comparator, outcomes, time points, setting) and characteristics (i.e. date of last search, comprehensiveness, and quality) of all eligible systematic reviews. Where there are multiple reviews addressing the same research question, we will compare the review characteristics and exclude those which are “superseded by a later review, or (contain) no additional (studies) compared with a review of similar, or higher, methodological quality” [79, 94]. For example, an up-to-date, high-quality systematic review may report on a single intervention (e.g. acupuncture) while another review, of lower methodological quality and with an older search date, may report on a number of alternative therapies including acupuncture. Although superseded by the former in terms of quality and recency, the latter review captures evidence on additional interventions. Inclusion of both reviews would be necessary to capture all available information on alternative therapies for smoking cessation. In this particular example, we would rely on the former review for data on acupuncture and on the latter for all other interventions (i.e. excluding acupuncture). As described by Pollock et al., the decision to exclude reviews based on these criteria can be a complex process often due to slight differences in research questions [94]. The criteria above will be used as a guide; with the pool of candidate reviews in hand, information will be mapped to facilitate decisions about potential exclusion. Decisions to exclude reviews due to redundancy will be tracked and documented in a table of characteristics of excluded reviews.

In cases where overlapping data cannot be avoided (i.e. overlapping reviews with similar search dates, quality, and comprehensiveness), we will include overlapping reviews and calculate the degree of overlap using the corrected covered area (CCA) [83, 93]. Although reporting the degree of overlap is recommended, it does not minimize or omit potential bias caused by inclusion of overlapping reviews [83, 93]. The CCA is calculated using the formula below, where *N* is the total number of studies across reviews (including multiple occurrences of the same study), *r* is the number of unique (first occurrence) studies, and *c* is the number of reviews.

$$ CCA=\frac{N-r}{rc-r} $$

The benefit of the correction for primary studies is that it diminishes the impact of large reviews that may add area but not necessarily overlap. Hence, the CCA corrects for the first time that studies are counted. The higher the CCA value, the greater the overlap among reviews: CCA value 0–5 would represent slight overlap, 6–10 of moderate overlap, 11–15 of high overlap, and > 15 of very high overlap.

Mapping of review characteristics will be conducted by a single reviewer. The decision to exclude a review, using the criteria described above, will be made by two reviewers via discussion, with review by the guideline WG. Where overlapping reviews are included, concordance of results/conclusions will be explored (see the “Discordance” section of the manuscript).

#### Quality assessment of systematic reviews

The methodological quality of reviews will be evaluated according to the AMSTAR 2 instrument (Additional file 3). This updated version of the original AMSTAR tool allows for the appraisal of systematic reviews of randomized and non-randomized studies of interventions [95]. We will evaluate each review against the 16-item instrument. An overall rating of quality will be assigned according to the algorithm suggested by Shea et al. [95]. Reviews failing to meet any of the seven critical AMSTAR 2 items will be deemed to have a “critical flaw” while non-fulfillment of the remaining items will be deemed a “non-critical weakness” of the review (Additional file 4). Reviews with one or more critical flaws will receive a low or critically low rating, respectively. Reviews with no critical flaws will be considered either high or moderate quality depending on the number of non-critical weaknesses (i.e. high-quality reviews have a maximum of one non-critical weaknesses and moderate-quality reviews have more than one weakness). Aside from decisions on inclusion related to assessing duplicate or overlapping reviews, reviews will not need to meet a particular threshold for methodological quality to be included.

The quality of systematic reviews will be evaluated by one reviewer and verified by another. Disagreements regarding by-item and overall rating of quality will be resolved by consensus or third-party adjudication if consensus cannot be reached.

#### Data extraction and management

Data extraction forms will be developed a priori in DistillerSR and pilot tested on a sample of studies to adjust forms, where needed, to maximize efficiency. Full data abstraction will be completed by one reviewer and verified by a second reviewer. Disagreements will be resolved by consensus or third party adjudication if consensus cannot be reached.

Additional file 5 lists draft items to be collected from reviews during data extraction. We will extract data as synthesized and/or reported in the reviews. We will not consult primary studies for the purpose of data extraction, risk of bias assessment, or for verifying the accuracy of the data reported in the systematic reviews.

We will collect data regarding outcomes of interest as reported by review authors. For reviews reporting a meta-analysis, we will collect the pooled effect estimates, corresponding confidence intervals, and results of statistical tests for heterogeneity (e.g. number of studies, number of participants, chi-square, Cochrane Q, corresponding *p* values, *I*^2^).

For network meta-analyses, ideally sufficient evidence from direct comparisons will be available, and treatment effect estimates along with measures of uncertainty from those analyses will be extracted. However, where little to no evidence from direct comparisons is available and indirect comparison data exist, we will extract both analyses and determine extent of consistency of results and make appropriate interpretations. For indirect comparison analyses, effect estimates and corresponding credible intervals will be collected from indirect comparisons. We will extract and transparently describe if and how authors’ ranking of treatments was used, ensuring appropriateness; ranking may take the form of rank probabilities, mean/median rank, surface under the cumulative ranking (SUCRA) curve, or a P-score [96–98].

For outcomes where a pooled analysis was not performed, how data are extracted will be informed by authors’ reporting. For example, if effect estimates from primary studies are reported, then a range of those effects could be extracted. In the absence of optimal quantitative data, a narrative summary of findings will be extracted from the reviews. Data will be collected for all reported and relevant (see Table 1) time points of follow-up.

Where reviews partially overlap with the scope of interest, such that a subset of studies may be conducted in a different population (e.g. adolescents), setting (not relevant to primary care), or other relevant parameter, we will attempt to determine whether the analyses undertaken are sufficiently direct to the overview question by considering the relative contribution of those studies to the analysis, subject to adequate reporting of this information. How these analyses are handled (inclusion versus exclusion) will be reviewed with the WG for their input; those decisions and any accompanying uncertainty in the applicability of the included results will be detailed in the report.

#### Subgroup analysis

The overview will seek information on various factors that would typically be considered variables for effect modification. In the case of an overview, we expect to encounter reviews that have undertaken subgroup or meta-regression analyses. There may also be reviews through the process of defining scope that would have focused their interest according to a particular factor, such as evaluating the effects of an intervention in a particular setting. Reviews addressing both of these approaches will be included. Variables of interest listed below are those that we have considered as being potentially important effect modifiers that would influence the development of guideline recommendations or implementation considerations. According to guidance, we have restricted subgroup analysis to characteristics that are measured at baseline rather than after randomization [99].

Populations

- Fewer versus more quit attempts (specific groupings will depend on what is found in the literature)
- Opportunistic versus individuals seeking treatment
- Baseline level of nicotine dependence (e.g. using a validated scale or cigarettes per day as a proxy)
- By demographic factors (age, SES, sex, ethnicity, LGBTQ+)
- By comorbid conditions (e.g. mental illness, HIV infection, cardiovascular disease, COPD, obesity, substance use disorder)
- By pregnancy status

Intervention-related variables

- Dose, type, duration, number of sessions
- Specific forms of an intervention (e.g. yoga as a form of exercise)
- KQ1a/b: behavioural change technique (e.g. providing information on consequences of smoking, explaining the importance of abrupt cessation, receiving prompt commitment from the patient)

Settings

- Family medicine clinics
- Walk-in clinics
- Smoking cessation clinics
- Urgent care facilities
- Emergency departments
- Public health units
- Pharmacies
- Dental offices
- Behavioural health/substance use treatment facilities (ambulatory or outpatient)
- Telehealth
- Academic research settings

Other variables

- By industry funding status (subgroup and/or sensitivity analyses performed in eligible reviews will be sought)

#### Evidence synthesis

While there are both simple (e.g. comparing 95% confidence intervals, statistical test of summary estimates) and complex (e.g. Bucher method, network meta-analysis) methods available for indirect comparisons of treatments across reviews, all approaches are based on the assumption that the primary studies are similar [85, 100]. This would require overview authors to be familiar with the primary study literature and not to rely solely on review authors’ reporting of the primary studies [85]. Given that we will not have opportunity to read and become familiar with the primary study reports themselves, conducting network meta-analyses or informal indirect comparisons of interventions will not be performed. As noted above, any existing network meta-analyses located in the literature will be included and commented on.

Similarly, subgroup analyses within reviews will provide evidence for effect modification. For factors that comprise the focused scope of a given review, as described in the previous section, we will provide the appropriate statements relating to interpretation but be unable to perform comparisons across reviews in the absence of the direct familiarity with the primary studies. Where possible, we will evaluate the credibility of subgroup analyses [99, 101, 102].

Although a narrative synthesis of available evidence to ensure appropriate interpretation will be provided for readers, the use of GRADE tables will facilitate appropriate presentation of this information in tabular form to avoid juxtaposition that may lend to inappropriate comparisons on the part of the reader [83, 85, 103]. Comparisons across reviews with similar scope will be limited to an assessment of the extent of concordance or discordance of the review results and, for discordance, an exploration of a potential explanation.

#### Discordance

Reviews that overlap in terms of scope may present discordant results and/or conclusions due to variation in eligibility criteria, data extraction, risk of bias assessment, data synthesis approach, or interpretation of the results [104]. In those instances, we will investigate the source(s) of discordance using the algorithm developed by Jadad et al. as a guide [104, 105].

Where overlapping reviews of similar quality rely on the exact same studies, we will investigate whether discordance was due to differences in data extraction (e.g. reviews may have extracted data at different time points of follow-up or reviews may vary regarding definitions of outcomes or outcome measurement methods), heterogeneity testing (e.g. reviews differ in their investigation of clinical and methodological heterogeneity and the decision in which to conduct a meta-analysis), or the synthesis approach (e.g. quantitative versus qualitative synthesis or in the statistical methods used).

If overlapping reviews do not rely on the exact same studies, we will investigate differences in the eligibility criteria. If similar, we will evaluate whether discordance is attributable to differences in the search strategies (e.g. number and type of databases searched, whether grey literature was searched) or in the application of the eligibility criteria. If reviews use different eligibility criteria, Jadad et al. [105] recommend comparing the publication status of primary studies (e.g. whether there are differences in the inclusion of unpublished reports), evaluation of the methodological quality of primary studies (e.g. differences across reviews regarding the assessment of quality of primary studies and how quality was used in interpreting the results of the review), language restrictions, and quantitative synthesis [105].

In addition to exploring sources of discordance, we will categorize discordance as follows: (1) direction of effect (i.e. reviews report results in opposite directions), (2) magnitude of effect (i.e. reviews report results in the same direction but differ in the size of the effect estimate), and (3) statistical significance (i.e. statistical significance reached in one review but not others) [105].

#### Quality of the body of evidence

The Task Force endorses the use of GRADE methodology for assessing the quality of the body of evidence for critical and important outcomes [106]. Currently, there are no methods to evaluate the strength of evidence across systematic reviews [83]. For each outcome of interest reported in each individual review, we will provide GRADE assessments by intervention/comparison [107]. We will not evaluate the strength of the evidence across reviews.

For reviews that have used GRADE methods, we will provide results for the overall quality of evidence, including reasons for downgrading. If available, we will also report the ratings for each of the five domains of GRADE (i.e. risk of bias, imprecision, indirectness, inconsistency, publication bias). We will not consult primary studies as a quality control measure.

If GRADE methods were not used in a given review, we will attempt to conduct GRADE assessments using information available in the review (e.g. risk of bias assessments). This will likely be challenging due to reporting issues; therefore, we will provide our best interpretation based on the available information and note any limitations. For systematic reviews that include a network meta-analysis, using information reported in the review, we will evaluate the quality of evidence using the GRADE extension for network meta-analysis [108]. As above, we will not consult primary studies for the purpose of conducting GRADE assessments. We will make note if it is not possible to conduct GRADE for a given review or outcome.

### Stage 2: Updated systematic review on electronic cigarettes for smoking cessation

#### Literature search

The search strategy for this update will be developed using the search strategy of the candidate systematic review, once identified. The search strategy of the candidate review will be evaluated and modified as necessary. Databases will be searched from the last search date of the review. Using the OVID platform, we will search Ovid MEDLINE®, Ovid MEDLINE® Epub Ahead of Print, In-Process & Other Non-Indexed Citations, Embase Classic + Embase, and PsycINFO. We will also search the Cochrane Library on Wiley. The final search will be peer-reviewed using the PRESS 2015 guideline [86]. Results of the PRESS reviews will be provided in an appendix in the final report. The grey literature will be searched using the same approach outlined for the overview of reviews.

#### Eligibility criteria

Studies will be selected for inclusion using the criteria outlined in Table 3.

**Table 3:** Inclusion and exclusion criteria for an updated review on e-cigarettes

	Inclusion	Exclusion
Population	Adults (≥ 18 years) who are current tobacco smokers (as defined by a given study)	▪ Studies exclusively in children/adolescents (i.e. under 18 years old) ▪ Studies that involve interventions targeted to adults other than the tobacco smoker (e.g. partners, healthcare providers)
Intervention	• Nicotine or non-nicotine containing e-cigarettes^a^ • Nicotine or non-nicotine containing e-cigarettes combined with other smoking cessation treatment (behaviour and/or pharmacological)	Studies exclusively examining short-term use of nicotine or non-nicotine containing e-cigarettes (i.e. < 1 week)
Comparator	KQ2a: ▪ Non-nicotine containing e-cigarettes (i.e. placebo e-cigarettes) ▪ No intervention ▪ Usual/standard care ▪ Waitlist ▪ Minimal intervention KQ2b: • Alternative nicotine containing e-cigarettes (e.g. different generation e-cigarette or e-cigarette containing a different dose of nicotine) • Non-nicotine containing e-cigarettes • Other smoking cessation aids (e.g. nicotine replacement therapy)	Studies exclusively examining short-term use of nicotine or non-nicotine containing e-cigarettes (i.e. < 1 week)
Outcomes	Critical • Tobacco use abstinence (as defined in the study) Important • Reduction in tobacco smoking frequency/quantity • Relapse (KQ2b only)^b^ • Quality of life (using validated scales) • Adverse events (as defined in a given review) • Possible adverse outcomes: o Weight gain o Changes in emotional state (e.g. increases in anxiety, changes in mood, irritability) o Loss of social group^c^	
Timing of outcome assessment	For abstinence/relapse, and quality of life outcomes: Minimum 6 months from quit date (if reported) or from initiation of intervention (if quit date not specified) All other outcomes: • Any point after initiation of intervention	
Setting	Settings that could serve as the primary point of contact for individuals to receive smoking cessation advice, including: Family medicine clinics Walk-in clinics • Smoking cessation clinics • Urgent care facilities • Emergency departments • Public health units • Pharmacies • Dental offices • Behavioural health/substance use treatment facilities (ambulatory or outpatient) • Telehealth • Academic research settings	▪ Studies in settings not relevant to primary care including workplaces, schools, inpatient settings, and medical specialist settings ▪ Studies that take place in countries “high”, “medium”, or “low” on the Human Development Index http://hdr.undp.org/en/composite/HDI
Study design	For benefits: • Randomized controlled trials For harms: • Randomized controlled trials • Non-randomized controlled trials • Comparative observational study designs (e.g. prospective and retrospective cohort studies, case-control studies)	For benefits: • Non-randomized controlled trials • Observational study designs For harms: • Non-comparative studies • Cross-sectional studies For benefits and harms: • Systematic reviews • Case reports, case series • Editorials • Commentaries
Language	▪ English ▪ French	
Dates of publication	▪ Date of last search of the review to present date	

^a^Nicotine and non-nicotine containing e-cigarettes can serve as either an intervention or comparator

^b^The outcome “relapse” was initially considered critical based on WG rating. However, based on discussion with WG members, it was decided that this outcome should be considered important. It was also decided that this outcome is most important for KQ1b

^c^Although initially rated as being of limited importance by the WG, based on discussions with WG members, it was decided that this outcome should be considered as important. Clinical experts and patients rated this outcome as important

#### Study selection and data extraction

Study selection and data extraction will follow the same process described for the overview of reviews. Where study eligibility is unclear, authors will be contacted by email twice over 2 weeks for additional information.

We will collect both self-report and biochemically validated tobacco abstinence and relapse. Data will be collected for all reported and relevant (see Table 3) time points of follow-up. Where needed, we will convert data (e.g. standard error to standard deviation) to facilitate consistent presentation of results across studies. Authors will be contacted by email twice over 2 weeks if any information is missing or unclear. Refer to Additional file 6 for a list of draft items to be collected during data extraction

We will consult studies included in the original review to ensure that all outcomes of interest (Table 3) have been captured.

#### Risk of bias assessment

For consistency, risk of bias assessments/quality appraisal will be performed for all available studies (i.e. studies included in the original review and newly identified studies). The risk of bias of randomized and non-randomized controlled trials will be assessed by one reviewer using the Cochrane risk of bias (ROB) tool [109] (Additional file 7). We will consider industry funding under the “other sources of bias” domain of the tool. A modified version of the Scottish Intercollegiate Guidelines Network critical appraisal tool [110] (Additional file 8), which accounts for potential sources of bias including that arising from industry funding, will be used to evaluate the quality of prospective cohort studies. Verification will be done by a second reviewer. Disagreements will be resolved by consensus or third-party adjudication.

Some domains are outcome-specific and will be assessed at the outcome level. Overall risk of bias for the body of evidence will be evaluated according to the importance of domains, the likely direction of bias, and the likely magnitude of bias [109]. The Agency for Healthcare Research and Quality guidance will be followed for evaluating risk of bias for outcome and analysis reporting bias [111].

#### Analysis

Study characteristics will be summarized narratively and presented in summary tables. Where possible, relative and absolute effects with 95% confidence intervals will be calculated for the GRADE summary of findings and evidence profile tables. Risk ratios and risk differences will be used to report effects for dichotomous data. For calculating the risk difference from meta-analyzed data, we will use the median baseline risk for the control group in the included studies, although we may perform sensitivity analysis using differing baseline risks if thought to be suitable. For continuous outcomes, mean difference (i.e. difference in means) effect measures will be used for outcomes using the same measure and standardized mean differences for outcomes using different measures, consistent with GRADE guidance [112].

#### Meta-analysis

We will examine the extent of clinical and methodological heterogeneity to determine appropriateness of performing meta-analysis. The Cochrane’s Q (considered statistically significant at *p* < 0.10) and *I*^2^ statistic will be used to assess the statistical heterogeneity across included studies [113, 114]. If appropriate, data from the original systematic review will be meta-analyzed with data from newly identified studies, using random effects models. For time-to-event data, the hazard ratio will be pooled using the generic inverse variance method. Analyses will be stratified by study design. For observational studies, we will use adjusted risk estimates in the meta-analysis.

Should meta-analysis not be appropriate due to considerable heterogeneity, the range of effects will be presented and results will be discussed narratively. Studies will also be presented in a forest plot without a pooled risk estimate. Clinical and methodological sources of heterogeneity will also be explored using subgroup, sensitivity, and/or meta-regression analyses, depending on how data are reported in studies. We will follow previously published guidance for meta-regression [115].

#### Sparse binary data and studies with zero events

Results will be synthesized narratively if studies report rare events. The risk difference will be used for outcomes (e.g. serious adverse events) where at least one intervention group contains zero events.

#### Subgroup analysis

If there are sufficient data, the following subgroup analyses will be conducted:

- Fewer versus more quit attempts (specific groupings will depend on what is found in the literature)
- Opportunistic versus individuals seeking treatment
- Baseline level of nicotine dependence (e.g. using a validated scale or cigarettes per day as a proxy)
- By demographic factors (age, SES, sex, ethnicity, LGBTQ+)
- By comorbid conditions (e.g. mental illness, HIV infection, cardiovascular disease, COPD, obesity, substance use disorder)
- By use of other substances (alcohol, cannabis, opioids)
- By pregnancy status
- By setting (e.g. family medicine clinics, walk-in clinics, urgent care facilities)
- Nicotine content (groupings will depend on what is found in the literature)
- Intensity of behavioural therapy (groupings will depend on what is found in the literature)
- Duration of e-cigarette usage as part of the intervention (groupings will depend on what is found in the literature)
- By type or generation of e-cigarette device
- By industry funding

#### Sensitivity analysis

Sensitivity analyses restricted to low risk of bias studies may be performed. Sensitivity analyses may also be performed to explore statistical heterogeneity or to evaluate the impact of various decisions made during the conduct of the review.

#### Small study effects

To evaluate small study effects, a combination of graphical aids and/or statistical tests will be performed if there are at least 10 studies in the analysis.

#### Software

The Cochrane Review Manager software version 5.3 [116] will be used to conduct analyses. Where needed, Comprehensive Meta-Analysis (CMA) or Stata may be used.

#### Grading the quality of evidence and interpretation

For critical and important outcomes, the GRADE framework [106, 117] will be used to assess the quality of the evidence.

## Discussion

Smoking is a leading cause of preventable death and disability, accounting for nearly 20% of all deaths in Canada. It is estimated that the cost of tobacco use in Canada is around $16 billion CDN, when considering factors such as hospital expenditure, physician care, and economic losses associated with premature death and disability. In response to this important public health care issue, the Canadian Task Force on Preventive Health Care will be developing a national tobacco smoking cessation guideline informed by an overview of systematic reviews of the benefits and harms of various stop smoking interventions for adults and relevant subpopulations, where available. This document has outlined the methods for undertaking the overview and an update of e-cigarette evidence for that overview.

## Additional files

Additional file 1: PRISMA Statement for Protocols (PRISMA-P) checklist. (DOCX 18 kb)

Additional file 2: Search strategy for the overview of reviews. (DOCX 16 kb)

Additional file 3: AMSTAR 2 Critical Appraisal Tool. (DOCX 77 kb)

Additional file 4: AMSTAR 2 critical domains for assessing overall rating of quality. (DOCX 14 kb)

Additional file 5: Draft data extraction items for the overview of reviews. (DOCX 13 kb)

Additional file 6: Draft data extraction items for the updated review of e-cigarettes for smoking cessation. (DOCX 12 kb)

Additional file 7: Cochrane risk of bias tool. (DOCX 29 kb)

Additional file 8: Modified SIGN methodology checklist for cohort studies. (DOCX 26 kb)

Additional file 9: Stakeholder feedback. (DOCX 34 kb)

## Abbreviations

CAN-ADAPTT: Canadian Action Network for the Advancement, Dissemination and Adoption of Practice-informed Tobacco Treatment
COPD: Chronic obstructive pulmonary disorder
E-cigarette: Electronic cigarette
HIV: Human immunodeficiency virus
KQ: Key question
NICE: National Institute for Health and Care Excellence
NRT: Nicotine replacement therapy
RCT: Randomized controlled trial
SES: Socioeconomic status
WG: Working group
