# Supplementary material for: Effectiveness of stop smoking interventions among adults: protocol for an overview of systematic reviews and an updated systematic review
Source: Syst Rev. 2019 Jan 19;8:28. doi: 10.1186/s13643-018-0928-x (PMC6339342; doi:10.1186/s13643-018-0928-x)
Supplement: Supplementary file 4 — AMSTAR 2 critical domains for assessing overall rating of quality. (DOCX 14 kb) [file 13643_2018_928_MOESM4_ESM.docx]

# Additional file 4. AMSTAR 2 critical domains for assessing overall rating of quality

**Critical domains**

1. Registration of the protocol prior to commencement of the review
2. Adequacy of the literature search
3. Justification for excluding individual studies
4. Assessment of risk of bias of included studies
5. Appropriateness of the meta-analytic methods
6. Consideration of risk of bias when interpreting results
7. Assessment of presence and impact of publication bias

**Overall rating of quality:**

High: No critical flaws and ≤1 non-critical weakness

Moderate: No critical flaws and >1 non-critical weaknesses

Low: One critical flaw with or without non-critical weaknesses

Critically low: More than one critical flaw with or without non-critical weaknesses

**AMSTAR 2 Citation:** Shea BJ, Reeves BC, Wells G, Thuku M, Hamel C, Moran J, Moher D, Tugwell P, Welch V, Kristjansson E, Henry DA. AMSTAR 2: a critical appraisal tool for systematic reviews that include randomised or non-randomised studies of healthcare interventions, or both. BMJ. 2017 Sep 21;358:j4008.
