# Supplementary material for: Effectiveness of stop smoking interventions among adults: protocol for an overview of systematic reviews and an updated systematic review
Source: Syst Rev. 2019 Jan 19;8:28. doi: 10.1186/s13643-018-0928-x (PMC6339342; doi:10.1186/s13643-018-0928-x)
Supplement: Supplementary file 5 — Draft data extraction items for the overview of reviews. (DOCX 13 kb) [file 13643_2018_928_MOESM5_ESM.docx]

# Additional file 5. Draft data extraction items for the overview of reviews

**General review characteristics:** Last author, year of publication, date assessed as ‘up to date’, number of included studies, study design of included studies

**Population:** Age, ethnicity, socioeconomic status, comorbid conditions (e.g., mental illness, HIV infection, cardiovascular disease, COPD), % fewer versus more quit attempts, % opportunistic versus individuals seeking treatment, % pregnant women, etc.

**Interventions:** Type of interventions and related details (e.g., dose, duration, number of sessions). For behavioural, include behavioural change technique.

**Comparators:** Type of comparator and related details (e.g., dose, duration, number of sessions)

**Outcomes:** Outcome, outcome definition, method of outcome measurement (e.g., subjectively versus objectively measured), timing of assessment

**Setting:** Type of setting (e.g., family medicine clinics, walk-in clinics, urgent care facilities)

**Quality/Risk of bias:** By domain/construct or as reported in reviews

**Analysis:** Method of analysis (e.g., statistical technique for meta-analysis or network meta-analysis)

**Results:**

Meta-analysis: pooled treatment effect estimates and corresponding measures of uncertainty, results of statistical tests for heterogeneity

Network meta-analysis: Treatment effect estimates and measures of uncertainty, ranking of treatments with corresponding measures of uncertainty

Reviews which do not report pooled estimates: Effect estimates from primary studies reported as a range, narrative summary of results

Subgroup analysis: Whether the subgroup analysis was planned *a priori*, effect estimates and corresponding measure of uncertainty for each subgroup

**Quality of the body of evidence:** For each outcome, rating of each GRADE domain and overall rating of confidence in the estimate of effect. If the body of evidence is rated using a system other than GRADE, details regarding the approach used and the results of the assessment will be collected.

**Limitations:** Limitations noted by systematic review authors or by the overview research team
