# Supplementary material for: Effectiveness of stop smoking interventions among adults: protocol for an overview of systematic reviews and an updated systematic review
Source: Syst Rev. 2019 Jan 19;8:28. doi: 10.1186/s13643-018-0928-x (PMC6339342; doi:10.1186/s13643-018-0928-x)
Supplement: Supplementary file 6 — Draft data extraction items for the updated review of e-cigarettes for smoking cessation. (DOCX 12 kb) [file 13643_2018_928_MOESM6_ESM.docx]

# Additional file 6. Draft data extraction items for updated review of e-cigarettes for smoking cessation

**Publication details**: authors, year of publication, language, publication status

**Study characteristics**: study design, methods, country, setting, sample size, number of centres [if applicable], study setting, duration of follow-up, source of funding

**Population characteristics:** age, ethnicity, intent to quit (% opportunistic versus individuals seeking treatment), number of quit attempts (% fewer versus more quit attempts), socioeconomic status, comorbid conditions (e.g., mental illness, HIV infection, cardiovascular disease, COPD), % pregnant women

**Details regarding intervention:** type of e-cigarette, nicotine dosage, duration of use

**Details regarding comparator**: type of comparator (e.g., no intervention, e-cigarette, alternative smoking cessation age), dosage (if applicable), duration of use (if applicable), number of sessions (if applicable)

**Outcomes of interest**: definitions, measurement methods, data as reported for entire study population and subgroups of interest, variables considered in confounding adjustment.
