# Supplementary material for: Effectiveness of stop smoking interventions among adults: protocol for an overview of systematic reviews and an updated systematic review
Source: Syst Rev. 2019 Jan 19;8:28. doi: 10.1186/s13643-018-0928-x (PMC6339342; doi:10.1186/s13643-018-0928-x)
Supplement: Supplementary file 7 — Cochrane risk of bias tool. (DOCX 29 kb) [file 13643_2018_928_MOESM7_ESM.docx]

# Additional file 7. Cochrane Risk of Bias Tool

1. **Selection bias domain**: Random sequence generation

🔿 Low risk

🔿 Unclear risk

🔿 High risk

Support for judgement:

1. **Selection bias domain**: Allocation concealment

🔿 Low risk

🔿 Unclear risk

🔿 High risk

Support for judgement:

1. **Performance bias domain**: Blinding of participants and personnel (for each outcome)

🔿 Low risk

🔿 Unclear risk

🔿 High risk

Support for judgement:

1. **Detection bias domain**: Blinding of outcome assessment (for each outcome)

🔿 Low risk

🔿 Unclear risk

🔿 High risk

Support for judgement:

1. **Attrition bias domain**: Incomplete outcome data (for each outcome)

🔿 Low risk

🔿 Unclear risk

🔿 High risk

Support for judgement:

1. **Reporting bias domain**: Selective reporting

🔿 Low risk

🔿 Unclear risk

🔿 High risk

Support for judgement:

1. **Other sources of bias (industry funding)**

🔿 Low risk

🔿 Unclear risk

🔿 High risk

Support for judgement:
