# Supplementary material for: Effectiveness of stop smoking interventions among adults: protocol for an overview of systematic reviews and an updated systematic review
Source: Syst Rev. 2019 Jan 19;8:28. doi: 10.1186/s13643-018-0928-x (PMC6339342; doi:10.1186/s13643-018-0928-x)
Supplement: Supplementary file 9 — Stakeholder feedback. (DOCX 34 kb) [file 13643_2018_928_MOESM9_ESM.docx]

**Additional file 9. Stakeholder feedback**

| **Stakeholder** | **(1) Do the research questions address the clinically important issues?**  **(2) Are there any important sources of evidence that we did not include that should be considered in our review?** | **Do you have any major concerns about the protocol that we should address?** | **Additional comments** | **Comments, Responses, and Changes to Manuscript (as applicable)** |
| --- | --- | --- | --- | --- |
| Stakeholder #1 | (1) Yes. Last update to national cessation guidelines (CAN-ADAPPT) was in 2012 and did not include vaping products, which have since become one of the favoured means by which some smokers wish to quit smoking. Update was needed to provide clinicians with most up-to-date advice. For the sake of clarity, the key questions could be more explicit about the fact that they are examining health-related benefits and harms (rather than examining these more broadly).  (2) Yes. North American Quitline Consortium – website (publications); Ottawa Hearth Institute (Ottawa Model for Smoking Cessation) | See comments in track changes Also Line 168: most e-cigarettes are battery-operated, but some are not. Suggest adding the word “most” in front of e-cigarettes.  Line 176: Tobacco and Vaping Products Act (plural, not singular) It is not clear whether this is a refresh of the 2012 CAN-ADAPPT guidelines or an entirely different new product. For those familiar with CAN-ADAPPT making this linkage more explicit might help setting this protocol in context | See comments in track changes. | **Last update to national cessation guidelines (CAN-ADAPPT) was in 2012 and did not include vaping products, which have since become one of the favoured means by which some smokers wish to quit smoking. Update was needed to provide clinicians with most up-to-date advice.**  Thank you.  **For the sake of clarity, the key questions could be more explicit about the fact that they are examining health-related benefits and harms (rather than examining these more broadly).**  Thank you for your feedback. All key questions specify that the intent of this work is to investigate the benefits and harms of tobacco cessation interventions. As we are examining outcomes which may not directly be related to health (e.g., behavioural outcomes such as tobacco cessation, quality of life factors), we have opted to keep the KQs broad and not add ‘health-related’. No changes made to the manuscript.  **North American Quitline Consortium – website (publications); Ottawa Hearth Institute (Ottawa Model for Smoking Cessation)**  Thank you for providing additional sources of evidence. Both have been added to our list of grey literature sources.  **Line 168: most e-cigarettes are battery-operated, but some are not. Suggest adding the word “most” in front of e-cigarettes.**  Thank you. The change has been made.  **Line 176: Tobacco and Vaping Products Act (plural, not singular)**  Thank you. The change has been made.  **It is not clear whether this is a refresh of the 2012 CAN-ADAPPT guidelines or an entirely different new product. For those familiar with CAN-ADAPPT making this linkage more explicit might help setting this protocol in context**  This evidence review will inform the development of a new guideline for the Canadian Task Force on Preventive Health Care. This is reported in the ‘Objectives and Key Question’ section of the manuscript. It is unrelated to the CAN-ADAPPT guidelines.  **Line 103: this is not an annual figure. It refers to 2012 only.**  Thank you. As suggested, we have removed ‘annually’ from the text.  **Suggest sticking to either CCHS or CTADS/CTUMS as a data source since they have different underlying smoking prevalence.**  To our knowledge, neither source provides data on smoking rates by both education and income which is why we relied on both sources. No changes made to the manuscript.  **Data from CCHS, which is not on reserve; however, on-reserve estimate from RHS 2015/16 is 40% therefore in line with the range presented here.**  Thanks for this information. As the on-reserve estimate falls within the range presented, no changes made to the text.  **Smoking estimate for Newfoundland and Labrador: 18%**  Where possible, we have updated the statistics reported in the background section using the recently released 2017 Canadian Tobacco, Alcohol and Drugs Survey. The rate of smoking in Newfoundland and Labrador is 20%.  **This is ever use of an e-cigarette, which will almost inevitably go up – once someone has tried, they will remain an ever user until aging out of the cohort or dying. A more accurate measure of use would be past-30-day or other measure of current use.**  In response to journal peer-review feedback, we have replaced prevalence of use data with the proportion using e-cigarettes as a cessation aide. This better aligns with the intent of the paragraph.  **It would be helpful to have a definition(s) for smoking cessation.**  A definition has been added to the background section.  **Quitlines offer a larger range of interventions, including reactive and proactive counselling by phone or online. Interactive web-based cessation support (instant chat, tailored emails, social media groups, etc) have increased in popularity in recent years, and there is a lack of guidance on their use.**  Thank you for clarifying that quitlines can be proactive or reactive forms of counselling. We have edited the sentence accordingly (i.e., to remove quitlines as an example of reactive counselling only). Both telephone and internet (self-help and those involving counselling support) based interventions will be investigated.  **Public Health England guidance document “Stop smoking options: guidance for conversations with patients” includes e-cigarettes. Published Aug 20, 2018.**  Thank you. We have edited this section to include this guidance.  **Self-help and technology-based interventions are not delivered by a primary care practitioner. Will they be excluded?**  Interventions that can be delivered or referred to by primary care practitioners will be included. Because primary care practitioners can refer patients to self-help or web-based interventions, these will be included.  **Please also discuss the LGBTQ+ . Suggestion is to add LGBTQ+ as a population subgroup of interest (and perform subgroup analysis) for both the overview of reviews and the updated review on e-cigarettes.**  Thank you for your suggestion. We have added the LGBTQ+ as a subgroup of interest.  **Is there a benchmark for the intensity of counselling?**  A Cochrane systematic review on individual behavioural counselling for smoking cessation included eleven studies which compared intensive counselling to less intensive counselling. Studies varied in terms of number and duration of sessions offered to those in the high intensity group. For example, the number of sessions for the high intensity intervention ranged from 4 to 24 across studies. Given this variation, we have decided to define interventions broadly in terms of intensity and brevity (i.e., ‘very brief’ or ‘brief’ practitioner advice) rather than specifying a threshold a priori. We will rely on review authors’ definition of intensity.  **RE: Some of the new approaches to cessation (technology-based) occur outside the healthcare setting. How are they going to be captured?**  We are seeking reviews in which some or all of the included studies are in settings that could serve as the primary point of contact for individuals to receive smoking cessation advice, including family medicine clinics, walk-in clinics, smoking cessation clinics, etc. The interventions do not need to be delivered in these settings. For example, a primary care practitioner may refer the patient to interventions that could be delivered outside the primary care setting (e.g. self-help, telephone- or internet-based interventions). Reviews of such interventions would be eligible for inclusion.  **Addition of ‘telehealth’ as a primary care setting**.  Thank you. We have added telehealth as a setting of interest.  **Please provide an example. [*This comment is in regards to Table 2, ‘relapse (only when the comparator is an active intervention)’ as an outcome of interest*]**  Thank you for the suggestion. We have provided an example in the footnote section of Table 2 (KQ1c).  **Suggestion to include the following outcome: “improvement/change in health outcomes”**  Thank you for your suggestion. Given that the health benefits of successful tobacco cessation are very well established, the Working Group has decided to not examine these outcomes.  **I found this explanation very convoluted. My attempted less confusing explanation is below, but might it be easiest to include the actual formula? It’s hard to figure out the order of operations in the denominator from the text.**  **“The CCA calculates the total number of studies minus the number of unique studies (studies that are included in only one review). That value is then divided by the product, reduced by the number of unique studies, of the number of unique studies and the number of reviews.”**  Thank you. We have added the formula to the manuscript, including an explanation of the variables.  **Addition of a subgroup of patient seeking treatment to maintain abstinence.**  The focus of the overview of reviews and the updated review on e-cigarettes is current tobacco smokers. As such, patients seeking treatment to maintain abstinence would be outside the scope of this work. |
| Stakeholder #2 | (1) Yes. The research questions are definitely important issues, especially Key question 1c of Stage 1: What are the benefits and harms of behavioral change techniques or clusters of techniques to promote cessation of tobacco smoking among adult smokers.  Knowing the best behavioral change techniques will be very helpful for clinicians in addressing smoking cessation with their patients.  (2) No. | In Table 1, under the column interventions, interventions to promote abrupt tobacco smoking cessation will be considered. Have you thought about looking at gradual cessation interventions? Also, did you consider comparing different pharmacotherapies so that clinicians would be able to tell their patients which one are the most effective?  -At lines 337-339, it would be helpful to understand how patients identified through patient engagement activities conducted by the St. Michael's Hospital Knowledge Translation Program will also rate the outcomes.  - In Table 1, on what basis were Studies/reviews where >20% of included participants are under 18 years old excluded?  - In Table 1, in the comparator column of KQ1a, minimal intervention can be confused with minimal advice.  - In Table 1, it is not clear what countries are excluded on the Human Development Index: high, medium and low?  - In Table 3, for KQ1b, I do not understand why Non-nicotine containing e-cigarettes and alternative smoking cessation aids (e.g., nicotine replacement therapy) are used as comparators. If I understand correctly the question, the effectiveness of different electronic cigarettes will be compared: either different generation of e-cigarettes or different doses of nicotine in electronic cigarettes. NRT comparator should be used for the first question What are the benefits and harms of electronic cigarettes for tobacco smoking cessation in adults? It is already the case with non-nicotine containing e-cigarettes. | None | **The research questions are definitely important issues, especially Key question 1c of Stage 1: What are the benefits and harms of behavioral change techniques or clusters of techniques to promote cessation of tobacco smoking among adult smokers.**  **Knowing the best behavioral change techniques will be very helpful for clinicians in addressing smoking cessation with their patients.**  Thank you.  **In Table 1, under the column interventions, interventions to promote abrupt tobacco smoking cessation will be considered.** **Have you thought about looking at gradual cessation interventions?**  Thank you. We will consider interventions intended to promote gradual tobacco smoking cessation. The eligibility criteria in tables 1 and 2 have been updated accordingly.  **Also, did you consider comparing different pharmacotherapies so that clinicians would be able to tell their patients which one are the most effective?**  Yes, we are interested in reviews that compare pharmacotherapies. Systematic reviews may identify and include head-to-head trials (i.e., those that directly compare pharmacotherapies). Such reviews would be included in KQ1b. Network meta-analyses, which would also be eligible, would provide the best evidence as they would allow for indirect comparisons when head-to-head trials are unavailable.  **At lines 337-339, it would be helpful to understand how patients identified through patient engagement activities conducted by the St. Michael's Hospital Knowledge Translation Program will also rate the outcomes.**  Detailed methods used in Phase 1 projects are found in the CTFPHC’s Patient Engagement Protocol (http://canadiantaskforce.ca/methods/patient-preferences-protocol/). We have added a link within this protocol.  **In Table 1, on what basis were ‘Studies/reviews where >20% of included participants are under 18 years old’ excluded?**  The Canadian Task Force on Preventive Health Care has an existing guideline for the prevention and treatment of tobacco smoking in children and adolescents. The reviews outlined in this protocol are intended to inform a guideline for tobacco cessation in adults.  We have since decided to omit the 20% threshold for determining eligibility as (1) 20% is an arbitrary cut-off and, (2) through a scan of available reviews, we have realized that many reviews fail to adequately report the age of participants of the included primary studies making it difficult for us to determine whether the 20% threshold has been met. As such, we have altered the population eligibility criteria so that reviews (or studies in the case of the e-cigarette update) exclusively in children/adolescents will be excluded. Subject to adequate reporting, we will consider the contribution of the subset of studies including children/adolescents to a given analysis and then make decisions to include or exclude.  In relation to this, we have also added schools (e.g., elementary schools, high schools, colleges, universities) as ineligible settings.  **In Table 1, in the comparator column of KQ1a, minimal intervention can be confused with minimal advice.**  Thank you for pointing this out. We have chosen to keep ‘minimal advice’ as an interchangeable term for ‘very brief’ advice as both are used in clinical practice. While ‘minimal advice’ may be considered a form of ‘minimal intervention’, the latter is broader and may include other various forms of limited interventions. We will rely on how review authors define interventions and comparators and we plan to to provide specification, where reported.  A minor change has been made to Tables 1 & 2: ‘Very brief advice/minimal advice’ is now changed to ‘Very brief/minimal advice’.  **In Table 1, it is not clear what countries are excluded on the Human Development Index: high, medium and low?**  We will exclude reviews if more than 50% of the included studies take place in countries ranked ‘high’, ‘medium’, or ‘low’ on the HDI index. We have edited tables 1-3 to improve clarity.  **In Table 3, for KQ1b, I do not understand why Non-nicotine containing e-cigarettes and alternative smoking cessation aids (e.g., nicotine replacement therapy) are used as comparators. If I understand correctly the question, the effectiveness of different electronic cigarettes will be compared: either different generation of e-cigarettes or different doses of nicotine in electronic cigarettes. NRT comparator should be used for the first question What are the benefits and harms of electronic cigarettes for tobacco smoking cessation in adults? It is already the case with non-nicotine containing e-cigarettes.**  The intent of KQ2a is to examine the benefits and harms of electronic cigarettes compared to placebo e-cigarettes (i.e., non-nicotine containing e-cigarettes), no intervention, usual/standard care, waitlist, or minimal intervention. The intent of KQ2b is to determine the comparative effectiveness of e-cigarettes. Eligible comparators for KQ2b are ‘active’ interventions including: alternative nicotine containing e-cigarettes (i.e., of different generation or dose of nicotine) as compared to the intervention, non-nicotine containing e-cigarettes, and other smoking cessation aides (e.g., NRT). Given that NRT is an ‘active’ intervention, it will be considered a comparator of interest for the comparative effectiveness question (i.e., KQ2b).  For clarity, a minor change was made to the Comparator row of Table 3: ‘Alternative smoking cessation aids (e.g., nicotine replacement therapy)’ changed to ‘Other smoking cessation aides (e.g., nicotine replacement therapy)’. |
| Stakeholder #3 | (1) Yes. Have you considered the impact of Cannabis use? As this can be smoked, are you confident all studies are tobacco only? I know you are using tobacco as a search word, but using smoking as a search word could potentially bring up studies of cannabis and tobacco use.  (2) No. Cannot think of any. Looks to be quite comprehensive. | The use of the term “smoker” without putting tobacco first is contrary to the Ottawa model. It is a negative connotation to refer to people as smokers; instead, consider using the term tobacco user or use tobacco smoke consistently throughout. Otherwise no concerns. The use of meta-analysis is addressed satisfactorily. | None | **Have you considered the impact of Cannabis use? As this can be smoked, are you confident all studies are tobacco only? I know you are using tobacco as a search word, but using smoking as a search word could potentially bring up studies of cannabis and tobacco use.**  The focus of this work is smoked tobacco. We may identify studies where the focus is not on smoked tobacco (e.g., chewing tobacco, cannabis); these studies will be excluded. We will, however, consider the impact of cannabis and other substances (e.g., alcohol, opioids) on the effectiveness of e-cigarettes for tobacco smoking cessation using subgroup analysis (see ‘Subgroup Analysis’ section in Stage 2 of the manuscript).  **The use of the term “smoker” without putting tobacco first is contrary to the Ottawa model. It is a negative connotation to refer to people as smokers; instead, consider using the term tobacco user or use tobacco smoke consistently throughout.**  Thank you for your suggestion. We have edited the manuscript accordingly. |
| Stakeholder #4 | Not reported. | See ‘additional comments’ column. | You may wish to reword this title. It is 19 words long… and I’m not clear on what an “overview of systematic reviews” is.  Introduction talks about groups with high prevalence of smoking. Should those with mental health issues also be mentioned in the intro given the issues experienced by this group?  Also, in the intro, I recommend using the term Indigenous, rather than Aboriginal. On this topic… to be respectful of Indigenous people and their use of sacred, I recommend including a statement or two to clarify the difference. It’s mentioned in a bracket… but I think complexity and sensitivity to this issue warrants a sentence or two for clarification purposes.  While you describe the author guidelines that are published on the topic, I think you should emphasis why there is a need for this particular guideline. What are the gaps in existing guidelineS? Are the existing guidelines out of date? For a particular healthcare professional only? Do they have different research questions? Its not obvious to the reader.  There are conflicts of interests noted by authors. While there can be great value to industry’s contribution to the research community, financial relationships with industry could influence recommendations, even when safeguards are employed. Please describe how these individuals managed their potential conflicts of interest.  Page 27- mapping of review characteristics will be conducted by a single reviewer. (who? Please state initials. I imagine that person is bilingual and can read both English and English?) The decision to exclude a review will be made by two reviews… for transparency purposes, will you be documenting the justification for each exclusion?  I understand the “undesirable outcomes” selected. Will there be any discussion on their effects on health inequities? Nicotine dependence? Replacing smoking with other non-healthy behaviours?  Given the involvement between industry and research (eg. NRT, Pharmacotherapies, working group members having conflicts of interest, etc.) I would like to see risk of bias tool for RCT modified to include a specific field for “is bias due to funding or sponsorship likely?” | **You may wish to reword this title. It is 19 words long… and I’m not clear on what an “overview of systematic reviews” is**.  We thank you for your feedback. While we agree that the title is lengthy, we have adhered to the ‘Preferred reporting items for systematic review and meta-analysis protocols (PRISMA-P)’ which is endorsed by the Systematic Reviews journal. The PRISMA-P reporting guideline recommends that titles of systematic review protocols identify the report as a protocol of a systematic review or as an update of a previous systematic review, as appropriate. The length of the title is largely attributed to adherence to this criterion. No changes made to the title.  An overview of systematic reviews, also known as a ‘systematic review of systematic reviews’, synthesizes evidence from existing systematic reviews rather than primary studies.  **RE: Introduction talks about groups with high prevalence of smoking. Should those with mental health issues also be mentioned in the intro given the issues experienced by this group?**  Thank you. We have added this information to the background of the report.  **Also, in the intro, I recommend using the term Indigenous, rather than Aboriginal. On this topic… to be respectful of Indigenous people and their use of sacred, I recommend including a statement or two to clarify the difference. It’s mentioned in a bracket… but I think complexity and sensitivity to this issue warrants a sentence or two for clarification purposes.**  Thank you. We have replaced ‘Aboriginal’ with ‘Indigenous’ and have elaborated on the traditional use of tobacco by Indigenous people.  **While you describe the author guidelines that are published on the topic, I think you should emphasis why there is a need for this particular guideline. What are the gaps in existing guidelines? Are the existing guidelines out of date? For a particular healthcare professional only? Do they have different research questions? Its not obvious to the reader.**  Great suggestion. Text has been added to the manuscript to communicate the need for the proposed guideline.  **There are conflicts of interests noted by authors. While there can be great value to industry’s contribution to the research community, financial relationships with industry could influence recommendations, even when safeguards are employed. Please describe how these individuals managed their potential conflicts of interest.**  As part of our process to vet conflict of interest, Dr. Selby provided a detailed statement about his declared interests. With regards to grants or research support and activities as a principal or co-investigator, Dr. Selby has largely received funding through academic institutions, non-profits, as well as federal and provincial governmental agencies.  Grant funding received from pharmaceutical companies was through open competitive processes. One independent, unrestricted training grant for pharmacists and NPs on safe prescribing of smoking cessation medications was obtained from Pfizer Canada. This online course is based on guidelines, is accredited by the University of Toronto and had no involvement of the sponsor in anyway. Dr. Selby has received free drugs for some trials from pharma companies. For the largest trials, drugs are bought at a discounted price through a tender process managed and adjudicated by the hospital which trials were done. Honoraria for speaking engagements and healthcare provider training were received from pharmaceutical companies, but Dr. Selby has declared that he does not use slides provided by industry. Dr. Selby speaks to all medications regardless of sponsor and does not speak to only one medication. His presentations have included information on harms, and Dr. Selby’s tobacco treatment algorithm includes quitting without assistance and behavioural approaches in addition to medications. He publishes videos online that give an indication of his advice around medication use. He has consulted or advised a number of private companies directly or indirectly involved in tobacco cessation, as well as two pharmaceutical companies on matters related to tobacco cessation. Dr Selby belongs to a number of special interest groups which consist of professional and academic groups or societies and other federal or provincial government-affiliated groups. His spouse has consulted for a pharmaceutical company that does not develop smoking cessation products but does develop therapies for related conditions such as lung cancer and COPD. He does not accept any hospitality or conference travel grants from industry sources and does not accept funds from tobacco, alcohol or food industry sources.  Dr. Selby will serve as a clinical expert but will not be a voting member of the guideline development process. Clinical experts are also not consulted regarding their opinions on guideline recommendations.  Dr. Hutton reports consultancy fees from Cornerstone Research Group. He will provide methodological expertise regarding the conduct of the evidence review. He is not a voting member of the guideline development process and will not be consulted regarding his opinion on guideline recommendations.  **Page 27- mapping of review characteristics will be conducted by a single reviewer. (who? Please state initials. I imagine that person is bilingual and can read both English and English?) The decision to exclude a review will be made by two reviews… for transparency purposes, will you be documenting the justification for each exclusion?**  As the availability of reviewers may change between now and project initiation, we have not included initials of reviewers in the protocol. The initials of reviewer(s) involved in key stages of the review process (i.e., screening, mapping, data extraction, risk of bias assessments) will, however, be reported in the completed systematic review. Bilingual members of the review team will screen, map, extract, and appraise French language reviews/studies.  Yes, the decision to exclude studies will be documented. This is described in the “Data mapping and overlap detection” section of the manuscript.  **I understand the “undesirable outcomes” selected. Will there be any discussion on their effects on health inequities? Nicotine dependence? Replacing smoking with other non-healthy behaviours?**  Thank you for your question. We will collect data on the effect of interventions on desirable and undesirable outcomes listed in the protocol but will not consider the downstream consequences of these outcomes. Although not considered as part of the systematic review process, aspects related to health equity are considered in the development of guideline recommendations according to the GRADE Evidence to Decision Framework.  **Given the involvement between industry and research (eg. NRT, Pharmacotherapies, working group members having conflicts of interest, etc.) I would like to see risk of bias tool for RCT modified to include a specific field for “is bias due to funding or sponsorship likely?**  There currently is no consensus on how to address industry funding in the Cochrane risk of bias tool. In the absence of such guidance, we will consider industry funding under the ‘other risk of bias’ domain of the tool. We have edited the manuscript to communicate this. |
| Stakeholder #5 | (1) Yes.  (2) No. | The protocol appears very thorough and well thought out. The only comment that I would make would be with reference to settings—I did not see where anything would be drawn from settings such as Community Health Centers, or Nurse practitioner led clinics There is ample practice of smoking cessation at these sites. I did not know if they are captured. | None | **The protocol appears very thorough and well thought out.**  Thank you.  **The only comment that I would make would be with reference to settings—I did not see where anything would be drawn from settings such as Community Health Centers, or Nurse practitioner led clinics There is ample practice of smoking cessation at these sites. I did not know if they are captured.**  Great point. While we have provided examples of relevant settings, it is not an exhaustive list. Community Health Centers and NP led clinics would be captured in our current criteria. |
| Stakeholder #6 | (1) Yes. Excellent questions to start the research  (2) Yes. People usually start smoking as youth. Adults rarely start smoking randomly. | Is this document complete? I feel like I am left hanging at the end. Its not really clear on how these interventions form the findings in the tables. | None | **Excellent questions to start the research.**  Thank you.  **People usually start smoking as youth. Adults rarely start smoking randomly.**  The Canadian Task Force on Preventive Healthcare has an existing guideline on the prevention and treatment of tobacco smoking in children and youth. As such, the evidence review presented in this manuscript is restricted to interventions for promoting smoking cessation in adults.  **Is this document complete? I feel like I am left hanging at the end. Its not really clear on how these interventions form the findings in the tables.**  Thank you. The manuscript outlines a protocol for an overview of systematic reviews on smoking cessation interventions and an updated review on e-cigarettes. No findings are reported as the study has yet to be conducted. The enclosed tables outline the eligibility criteria of the review with respect to the populations, interventions, comparators, outcomes, timing, and settings of interest. |
| Stakeholder #7 | (1) No. I am supportive of the questions that you are asking for smoked products, but am concerned that smokeless tobacco (chewing tobacco, etc.) use is being omitted from the study. I am also concerned about the availability of well-designed studies concerning the effects of e-cigarettes.  (2) Yes. The focus on the available research literature is important for the success of the systematic review, but there may be sources of gray literature that will be eliminated from the study yet still have important findings. | No, it seems straight forward. | None | **I am supportive of the questions that you are asking for smoked products, but am concerned that smokeless tobacco (chewing tobacco, etc.) use is being omitted from the study.**  Thank you for your feedback. Given that the evidence for smokeless tobacco is limited and that the burden is far greater for smoked tobacco, the Working Group decided to limit the scope of the review to smoked tobacco.  **I am also concerned about the availability of well-designed studies concerning the effects of e-cigarettes.**  We will take the strength of the evidence, which includes an assessment of risk of bias, into account when drawing conclusions and recommendations regarding the effect of e-cigarettes. Finding of a lack of evidence is still an important finding.  **The focus on the available research literature is important for the success of the systematic review, but there may be sources of gray literature that will be eliminated from the study yet still have important findings.**  We will search the grey literature and include any relevant grey literature sources identified. We will not exclude studies on the basis of publication status. |
| Stakeholder #8 | (1) Yes  (2) No | Major concerns to be addressed:  1. The reviewers have noted that there are two different methodological approaches that are being used for achieving stage one (evaluating the effectiveness of stop smoking interventions among adults delivered in primary care setting) versus stage two (updated review on e- cigarettes use for smoking cessation). The authors have described using different approach /methodologies for the update on e-cigarettes, other than the use of systematic reviews. They have included randomized and non-randomized trials, organizational websites and trial registries including grey literature which may be a bias in the protocol. It is unclear why different methodological approaches have been used. It is suggested that the authors provide a rationale as to why this is the case, or we suggest that the e- cigarette section be removed from the review or else apply a standard methodological approach as in stage one.  2. It is important for the authors to be mindful when reviewing the study sponsorship of the studies/reviews, especially where Tobacco Industry may be involved. If possible, the analysis should clearly state whether such studies have been incorporated or excluded in their reviews. The analysis can be separated into two e.g. without such studies and one that includes them. In recent years, Tobacco industries and their non-medical allies have shown an increasing interest in conducting studies, while publishing results in scientific journals, from obvious marketing purposes. One example is the paper published by R. Polosa et al. at the BMC Public Health in 2011 that found the E-cigarettes were more effective than NRTs for smoking cessation. This Study was also funded by Phillip Morris.  3. It appears that the authors aim to evaluate methods for smoking cessation in primary care settings only. If this is the case, then it should be reflected in the title of the protocol. However, the reviewers have noted that this may not be the case for electronic cigarettes e.g. in line 60, E-cigarettes are mentioned on abstract, on the same note with various stop smoking interventions that are available - which is not supported by evidence at the moment, and might confound readers and searchers. The writer would consider mentioning the E-cigarettes a questionable tool for quitting smoking.  4. Line 437, Table 2. Inclusion and Exclusion Criteria for Key Question 1c. In: Intervention; Exclusion criteria – the authors should consider adding Relapse Prevention in their exclusion criteria which is another published technique while working with smokers. | General Comments  The protocol clearly states the objectives and key questions, stages involved, methods for gathering evidence and tools for evaluating the quality of the evidence, number of participants/reviewers /expert consultations involved and the consensus process. Overall goal of the evidence review is to determine the effectiveness of stop smoking strategies in adults.  The review will include the systematic reviews evaluating the effectiveness of stop smoking interventions among adults delivered in the primary care setting, the protocol clearly defines the targeted primary care settings, eligibility criteria of the interventions reviewed with a focus on interventions that are currently approved or available in Canada. The inclusion and exclusion criteria for the study population, nature of intervention, comparator and outcome indicators for each key question is well documented.  An updated review on electronic cigarettes will also be conducted. The aim is to examine the benefits and harms associated with electronic cigarettes. The protocol explains the inclusion and exclusion criteria for the controlled trials that will inform on the use of e-cigarettes for smoking cessation. Similarly, study selection, data extraction process, bias assessment and analysis have been clearly explained. The authors have defined a comprehensive search strategy dating from 2008 to current date from various online databases. Likewise, unpublished data/reports from international organizational websites and trial registries, grey literature (English and French) will be included in the review.  Minor comments for consideration.  1. Line 114- The authors would consider adding people with substance use or mental health problems as another subpopulation group with higher rates of smoking prevalence (although most statistics are based from large studies from US e.g. Lasser K et al., (2000) on smoking and mental illness). There may be no similar large population studies among Canadian population. However, mental illness and substance use has been highly prevalent among current smokers in Canada. (Refer to Kirst et al, 2013).  Kirst, M.,Mecredy,G.,& Chaiton ,M. (2013).The prevalence of tobacco use comorbidities in Canada. Canadian Journal of Public Health 104 (3), e210-e215.  2. Line 228 – The authors can consider adding S-adenosyl-L-methionine [SAM-e] in their review as an alternative therapy, this is a dietary supplement that may be used as an adjunct therapy for treatment of depression (CANMAT guidelines for Management of Adults with MDD: Section 5. Complementary and Alternative Medicine Treatment, 2016). It has also been tested elsewhere for smoking cessation. | **The reviewers have noted that there are two different methodological approaches that are being used for achieving stage one (evaluating the effectiveness of stop smoking interventions among adults delivered in primary care setting) versus stage two (updated review on e- cigarettes use for smoking cessation). The authors have described using different approach /methodologies for the update on e-cigarettes, other than the use of systematic reviews. They have included randomized and non-randomized trials, organizational websites and trial registries including grey literature which may be a bias in the protocol. It is unclear why different methodological approaches have been used. It is suggested that the authors provide a rationale as to why this is the case, or we suggest that the e- cigarette section be removed from the review or else apply a standard methodological approach as in stage one.**  Stage 1 of this work will involve an overview of systematic reviews on tobacco cessation interventions including pharmacotherapies, electronic cigarettes, behavioural support, and alternative therapies. An overview of systematic reviews was selected for stage 1 given the number of interventions of interest and the availability of systematic reviews. A rationale for conducting an overview of reviews has been added to the manuscript.  Stage 2 will involve an updated systematic review (of primary studies) on electronic cigarettes for smoking cessation. An updated systematic review on e-cigarettes is necessary given the increasing use of and evolving evidence base for this intervention. This rationale appears in the ‘Objectives and Key Questions’ section of the manuscript. We plan to update the most recent, comprehensive and high quality systematic review on e-cigarettes identified in the overview of reviews (stage 1).  **It is important for the authors to be mindful when reviewing the study sponsorship of the studies/reviews, especially where Tobacco Industry may be involved. If possible, the analysis should clearly state whether such studies have been incorporated or excluded in their reviews. The analysis can be separated into two e.g. without such studies and one that includes them. In recent years, Tobacco industries and their non-medical allies have shown an increasing interest in conducting studies, while publishing results in scientific journals, from obvious marketing purposes. One example is the paper published by R. Polosa et al. at the BMC Public Health in 2011 that found the E-cigarettes were more effective than NRTs for smoking cessation. This Study was also funded by Phillip Morris.**  Thank you. As noted in the manuscript, we plan on performing subgroup analysis by industry funding status (see ‘Subgroup analysis’ headings in the methods section for Stage 1 and Stage 2). For Stage 2, we will also consider industry funding in risk of bias/quality assessments. We will not exclude studies/reviews on the basis of industry funding.  **It appears that the authors aim to evaluate methods for smoking cessation in primary care settings only. If this is the case, then it should be reflected in the title of the protocol.**  We thank you for your suggestion. Given that we are considering interventions delivered in AND referred to from primary care, specifying ‘primary care’ in the setting may be misleading. No changes made to the title. Relevant settings are outlined in Tables 1-3.  **However, the reviewers have noted that this may not be the case for electronic cigarettes e.g. in line 60, E-cigarettes are mentioned on abstract, on the same note with various stop smoking interventions that are available - which is not supported by evidence at the moment, and might confound readers and searchers. The writer would consider mentioning the E-cigarettes a questionable tool for quitting smoking.**  Given that e-cigarettes may be used for cessation (approximately 32% of Canadians who attempted to quit tobacco smoking in 2017 used e-cigarettes), the Working Group has decided to consider this as an intervention of interest. We have not commented on the effectiveness of eligible interventions, including e-cigarettes, in this protocol as the planned reviews will yield this information.  **Line 437, Table 2. Inclusion and Exclusion Criteria for Key Question 1c. In: Intervention; Exclusion criteria – the authors should consider adding Relapse Prevention in their exclusion criteria which is another published technique while working with smokers.**  For KQ1c, we plan on excluding all reviews which examine behavioural change interventions rather than behavioural change techniques. This would include reviews on Relapse Prevention which fail to specifically examine the behavioural change technique components of the intervention. We have kept the exclusion criteria broad (i.e., reviews of any behavioural change interventions rather than techniques) rather than specifying all behavioural change interventions. No changes made to the manuscript.  **Line 114- The authors would consider adding people with substance use or mental health problems as another subpopulation group with higher rates of smoking prevalence (although most statistics are based from large studies from US e.g. Lasser K et al., (2000) on smoking and mental illness). There may be no similar large population studies among Canadian population. However, mental illness and substance use has been highly prevalent among current smokers in Canada. (Refer to Kirst et al, 2013). Kirst, M.,Mecredy,G.,& Chaiton ,M. (2013).The prevalence of tobacco use comorbidities in Canada. Canadian Journal of Public Health 104 (3), e210-e215.**  Thank you. We have edited the background section to note higher prevalence of smoking in those with substance use disorders and mental health issues. Subgroup analysis by comorbid conditions (e.g., mental illness, substance use disorders) is planned.  **Line 228 – The authors can consider adding S-adenosyl-L-methionine [SAM-e] in their review as an alternative therapy, this is a dietary supplement that may be used as an adjunct therapy for treatment of depression (CANMAT guidelines for Management of Adults with MDD: Section 5. Complementary and Alternative Medicine Treatment, 2016). It has also been tested elsewhere for smoking cessation.**  Thank you for your suggestion. We have added this as an intervention of interest. |
| Stakeholder #9 | (1) Yes. Cessation is an important topic for review. The addition of e-cigarettes will enhance the utility of the review.  (2) Yes. I wonder if you wished to include GINA 2018 guidelines re: tobacco smoke being a risk factor for development of asthma, especially for those exposed in utero. | I am curious about the decision to remove motivational interviewing. While it is an ‘added skill’, removing it from the review may cause the loss of a method of counseling that is effective. A meta-analysis indicated that the level of training does not necessarily influence the success of MI. Many schools are now teaching or introducing this method to their students prior to graduation – perhaps your review will promote this movement. The meta-analysis: Lundahl, Kunz, Brownell, Tollefson, Burke. A meta-analysis of motivational interviewing: Twenty-five years of empirical studies. Research on Social Work Practice. 2010; 20(2):137-160.   In your manuscript you use the term 'smoker'.  This is a label and defines the individual by his disease (Tobacco use disorder), similar to asthmatic, diabetic, COPDer etc.  While we often think of labels as racial, socio economic in nature, I encourage you to resist the use of labels period and look for other options that are more patient centred, e.g. person first language - people who use tobacco, individuals who smoke etc. | None | **Cessation is an important topic for review. The addition of e-cigarettes will enhance the utility of the review.**  Thank you.  **I wonder if you wished to include GINA 2018 guidelines re: tobacco smoke being a risk factor for development of asthma, especially for those exposed in utero.**  Thank you for your suggestion. We have added the GINA 2018 guideline as a reference to the background section to support the association between smoking and respiratory issues.  **I am curious about the decision to remove motivational interviewing. While it is an ‘added skill’, removing it from the review may cause the loss of a method of counseling that is effective. A meta-analysis indicated that the level of training does not necessarily influence the success of MI. Many schools are now teaching or introducing this method to their students prior to graduation – perhaps your review will promote this movement. The meta-analysis: Lundahl, Kunz, Brownell, Tollefson, Burke. A meta-analysis of motivational interviewing: Twenty-five years of empirical studies. Research on Social Work Practice. 2010; 20(2):137-160.**  Thank you for your suggestion. After careful consideration, the Working Group has decided to exclude interventions that would require primary care practitioners to receive specialized training.  **In your manuscript you use the term 'smoker'.  This is a label and defines the individual by his disease (Tobacco use disorder), similar to asthmatic, diabetic, COPDer etc.  While we often think of labels as racial, socio economic in nature, I encourage you to resist the use of labels period and look for other options that are more patient centred, e.g. person first language - people who use tobacco, individuals who smoke etc.**  Thank you. We have edited the manuscript using patient-centered language. |
